# Calcium Activity Dynamics Correlate with Neuronal Phenotype at a Single Cell Level and in a Threshold-Dependent Manner

**DOI:** 10.3390/ijms20081880

**Published:** 2019-04-16

**Authors:** Sudip Paudel, Eileen Ablondi, Morgan Sehdev, John Marken, Andrew Halleran, Atiqur Rahman, Peter Kemper, Margaret S. Saha

**Affiliations:** 1 Department of Biology, College of William and Mary, Williamsburg, VA 23185, USA; spaudel@email.wm.edu; 2 Department of Biological Chemistry and Molecular Pharmacology, Harvard Medical School, Harvard University, Boston, MA 02115, USA; efablondi@g.harvard.edu; 3 Harvard Medical School, Harvard University, Boston, MA 02115, USA; msehdev@email.wm.edu; 4 Department of Bioengineering, California Institute of Technology, Pasadena, CA 91125, USA; jmarken@caltech.edu (J.M.); halleran@caltech.edu (A.H.); 5 Department of Computer Science, College of William and Mary, Williamsburg, VA 23185, USA; mrahman@email.wm.edu (A.R.); kemper@cs.wm.edu (P.K.)

**Keywords:** calcium, development, embryo, nervous system, neural development, calcium activity, *Xenopus*, sox2, tubb2b, Ca^2+^

## Abstract

Calcium is a ubiquitous signaling molecule that plays a vital role in many physiological processes. Recent work has shown that calcium activity is especially critical in vertebrate neural development. Here, we investigated if calcium activity and neuronal phenotype are correlated only on a population level or on the level of single cells. Using *Xenopus* primary cell culture in which individual cells can be unambiguously identified and associated with a molecular phenotype, we correlated calcium activity with neuronal phenotype on the single-cell level. This analysis revealed that, at the neural plate stage, a high frequency of low-amplitude spiking activity correlates with an excitatory, glutamatergic phenotype, while high-amplitude spiking activity correlates with an inhibitory, GABAergic phenotype. Surprisingly, we also found that high-frequency, low-amplitude spiking activity correlates with neural progenitor cells and that differentiating cells exhibit higher spike amplitude. Additional methods of analysis suggested that differentiating marker *tubb2b*-expressing cells exhibit relatively persistent and predictable calcium activity compared to the irregular activity of neural progenitor cells. Our study highlights the value of using a range of thresholds for analyzing calcium activity data and underscores the importance of employing multiple methods to characterize the often irregular, complex patterns of calcium activity during early neural development.

## 1. Introduction

Calcium serves as a pervasive and essential signaling molecule in virtually all biological systems [1,2,3]. To transduce calcium signals, organisms have evolved an array of calcium-dependent signaling pathways that relay amplitude- and frequency-based signals to regulate a wide array of physiological processes [4,5,6,7]. These include fertilization and egg activation [8,9,10], cell proliferation [11,12], differentiation [13], motility and migration [14], morphogenesis [15], autophagy and apoptosis [16], synaptic plasticity, senescence [17], and neurotransmission [17,18,19]. While calcium activity has been most extensively characterized in the mature nervous system [20,21], a growing body of work has provided evidence for an early and essential role for calcium in the developing nervous system [22,23,24,25,26].

Specific patterns of calcium activity have been implicated in many distinct phases of neural development, ranging from neural induction and neurotransmitter fate determination to neurite extension and neural tube closure [18,27,28,29,30,31]. These processes rely on the ability of calcium-dependent signaling pathways to encode amplitude and frequency-based signals that trigger responses from downstream effectors, which in turn regulate gene expression [7,22,32]. Notably, there is compelling evidence demonstrating that deregulation of these processes is associated with an array of human disease syndromes (reviewed in [33]).

Although calcium activity, particularly during embryonic development, can be observed across a vast continuum of different frequencies, amplitudes and patterns, it is typically divided into two broad categories: spikes and waves [34,35,36]. Waves are defined as transient increases in cytosolic calcium followed by a quick succession of similar increases propagated within the same cell or across many neighboring cells. Because intracellular waves are readily identifiable, they have been relatively well characterized, with established roles in egg activation, cell migration, and proliferation [8,14]. Spikes, in contrast, are canonically described as fast-rising, transient increases in cytosolic calcium that occur in relatively isolated single cells [34]. In the adult nervous system, stereotypical dynamics make spikes easily discernable. However, in the developing nervous system, spikes frequently lack clear periodicity and pattern, display low amplitudes, and often appear stochastic in nature, making them difficult to distinguish from background activity. This irregular behavior has resulted in a lack of consensus on how to identify a spike, with inconsistent criteria to define spiking activity applied throughout the field [37,38,39,40].

Despite the less regular nature of early calcium behavior and the difficulty of unambiguously identifying what constitutes a spike, this type of calcium activity is of undisputed importance. Spiking behavior in neural development is present in a wide array of vertebrate species and has been implicated in the processes of significance for human health, most notably in neurotransmitter phenotype determination [18,25,28,29]. Previous work has shown that at the early neurula stages elevated levels of spiking promote GABAergic (i.e., inhibitory) neuronal fates, while lower levels of spiking promote glutamatergic (i.e., excitatory) fates [18,28,29,41]. However, despite the clear importance of this specific type of calcium activity, many unanswered questions remain regarding the nature of calcium spikes during the critical neurula stages of vertebrate embryogenesis. For example, given that most studies examined calcium activity in broad regions of the embryo, it is not known whether the correlation between calcium spiking and neurotransmitter phenotype is valid at the level of single cells or only at population level. Additionally, the degree to which these patterns of calcium activity are cell-autonomous or -dependent on the maintenance of cell–cell interactions remains unknown.

To address these questions and further characterize spiking behavior during early vertebrate neurogenesis, we employed *Xenopus laevis*, an amphibian that has served as the classic model system for early neural development in vertebrates [24,42]. *Xenopus laevis* is particularly amenable for this set of experiments due to the availability of early-stage embryos and the accessibility of neural tissue at relevant stages of development. Additionally, cell culture experiments allowed calcium activity to be assessed under defined and reproducible media conditions. We focused our study on three specific questions. First, we asked if there is a correlation of neurotransmitter phenotype with specific patterns of calcium activity on the level of individual cells. To this end, we also investigated whether there was any correlation between calcium spiking activity and the earlier, and even more fundamental, developmental decision point of whether a cell maintains a neural progenitor state or undergoes differentiation. Secondly, we assessed the degree to which calcium activity and its association with specific gene expression is cell-autonomous, that is, whether cells isolated from their neighbors displayed patterns of calcium activity in vitro. Finally, given that embryonic calcium activity does not display the stereotypical patterns characteristic of mature neurons, and given the wide diversity of ways a spike has been defined in the literature, we asked if using different methods of analysis of calcium activity could lead to different experimental conclusions, a question that has important implications for our understanding of calcium activity in early neural development.

## 2. Results

### 2.1. Overview of Experimental Plan

**Figure 1:**
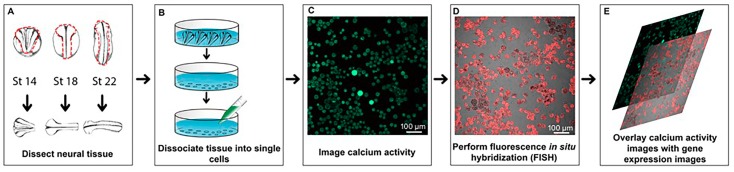
Overview schematic showing dissociation of neural tissue, calcium imaging and subsequent molecular phenotype identification of individual cells. (**A**) Dissection of embryonic neural tissue at neural plate (Stage 14), neural tube (Stage 18) and early tailbud stage (Stage 22). (**B**) Dissociation of explant in calcium and magnesium free solution followed by Fluo-4 AM treatment. (**C**) A representative fluorescence still image acquired using Nikon A1R T*i* Laser Scanning Confocal Microscope. (**D**) Identification of molecular phenotype using fluorescence in situ hybridization assay. (**E**) Overlay of calcium activity images with gene expression.

As discussed in the Introduction, previous studies have suggested that the decision between inhibitory and excitatory cell fates is correlated with and influenced by the frequency of calcium spikes, with elevated levels of calcium activity increasing the number of inhibitory neurons and lower levels of spiking resulting in more excitatory glutamatergic and cholinergic neurons [25]. To test whether neurotransmitter phenotype is correlated with calcium activity on the level of single cells, we performed time-lapse calcium imaging on dissociated embryonic neural tissue of *Xenopus laevis* at neural plate (Stage 14), neural tube (Stage 18) and early tail-bud (Stage 22) stages. To associate calcium activity unambiguously with specific cells, it is essential to have a means of delineating the cell boundaries and a means of tracking the cells, as significant cell movement occurs even during a 30-min span during these stages of development, both in vivo and in vitro. Given cell movement, we employed tracking software to ensure that we could precisely identify each cell, and then analyzed calcium activity using multiple methods including spike counting, power, entropy, and Hurst exponent analysis. To assess the phenotype of cells at the molecular level, fluorescent in situ hybridization (FISH) was performed using one of four different probes: *gad1.1* (glutamic acid decarboxylase 1) as a marker for inhibitory neurons, *slc17a7* (vesicular glutamate transporter 1, known as solute carrier family 17a member 7) as a marker for excitatory neurons, *sox2* (Sry-related HMG factor) as a marker for neural progenitor cells, and *tubb2b* (Neural Beta Tubulin) as a marker for neuronal cells committed to differentiation. To conduct a comprehensive analysis and to resolve some of the discrepancies in the literature, we investigated whether calcium activity correlated with the molecular phenotype in three different ways. First, we asked whether levels of calcium activity correlated with the actual levels of marker gene expression by performing both linear and nonlinear correlation analyses. Second, we analyzed whether cells that were positive for a given marker gene showed significantly different calcium activity compared to cells that were negative for that particular marker gene. Finally, we analyzed whether pairs of genes that are typically (although not exclusively) expressed in a mutually exclusive fashion (e.g., *gad1.1* and *slc17a7*) showed significantly different levels or patterns of calcium activity. Figure 1 presents a schematic overview of the experimental approach.

### 2.2. The Intensity of Gene Expression for gad1.1 and slc17a7 is not Directly Correlated with the Metrics of Calcium Activity on a Single-Cell Level

To determine whether the intensity of gene expression correlates with calcium activity in individual cells, we performed correlation analyses for both *gad1.1* and *slc17a7* using spike counting (to assess the frequency of spikes), average power, Hurst exponent estimation, and Markovian entropy measurements (to assess the periodicity, persistency, and predictability of the calcium activity dynamics). We correlated cellular calcium dynamics as quantified by each of these measures with the levels of expression of the marker genes quantified as a FISH intensity score by performing both a linear Pearson correlation analysis and a nonlinear correlation analysis as described above. No significant correlation was observed between calcium activity and the intensity of fluorescence signal for either *gad1.1* or *slc17a7* by any of the metrics we used (Tables S1 and S2). As baseline information for marker gene expression, we also performed a Kruskal–Wallis test to determine whether the percentages of cells expressing either *gad1.1* or *slc17a7* at the various FISH levels are similar across the three developmental stages. While the percentage of cells displaying *gad1.1* expression gradually increases from neural plate to tailbud stages, *slc17a7* increases initially and then decreases by tailbud stages.

### 2.3. At Neural Plate Stages, gad1.1-negative Cells Show more Entropic Low-amplitude Calcium Activity while gad1.1 Positive Cells Correlate with more Regular, Higher-Amplitude Activity

Although the intensity level of gene expression did not correlate with the metrics of calcium activity, we hypothesized that a cell’s exact level of gene expression may be less meaningful than a binary metric of whether that gene expression meets a certain threshold for biological relevance. We therefore tested whether calcium activity was significantly different in cells that were positive for a given neurotransmitter marker versus those that were negative for the same marker. As described in the Section 4, while we utilized 200% of the baseline fluorescence value as the threshold for a cell to be considered positive for expression of each gene of interest, we analyzed a range of gene expression thresholds to reduce the bias implicit in setting a single cutoff threshold (all values are presented in the Supplementary Materials). When using spike counting as the assay for calcium activity, there was no significant difference in *slc17a7*-positive and *slc17a7*-negative cells at any stage for any of the comparisons. However, when comparing *gad1.1*-positive cells with *gad1.1*-negative cells at the neural plate stage (Stage 14), *gad1.1*-negative cells showed significantly more spiking at lower-amplitude thresholds for spike definition than *gad1.1*-positive cells (Figure S1D), while at higher-amplitude thresholds (200% and 300% of baseline calcium activity), *gad1.1*-positive cells showed significantly more spiking activity than *gad1.1*-negative cells (Figure S1D). Consistent with these results, *gad1.1*-negative cells were characterized by greater entropy (less predictability) and *gad1.1*-positive cells showed greater Hurst scores (more persistent dynamics) at neural plate stages (Stage 14) (Figure S1). Using average power to analyze calcium activity at the neural plate stage did not reveal any significant differences between positive and negative cell populations (Table S3). However, at neural tube stages (Stage 18), average power scores showed mixed bimodal results, with both *gad1.1*-negative and *gad1.1*-positive cells having statistically significant higher average power scores (Figure S7C). This finding suggests that Stage 18 may be a transition to the early tailbud stage, at which stage *gad1.1*-positive cells display higher power (periodicity).

### 2.4. Slc17a7-positive Cells Exhibit Higher Levels of Low-amplitude Spiking than gad1.1-positive Cells, whereas gad1.1-positive Cells Exhibit Higher Levels of high-amplitude Spiking than slc17a7-positive Cells

Based on similar comparisons reported in the literature, our final analysis for analyzing neurotransmitter phenotype and calcium activity on a single-cell level entailed testing whether there were statistically significant differences between calcium activity in cells positive for the *gad1.1* inhibitory marker when compared to *slc1717*-positive excitatory cells. As described in the previous section, we used 200% of the background signal as the threshold to consider a cell positive for expression of a gene of interest, although data were analyzed at multiple thresholds.

**Figure 2:**
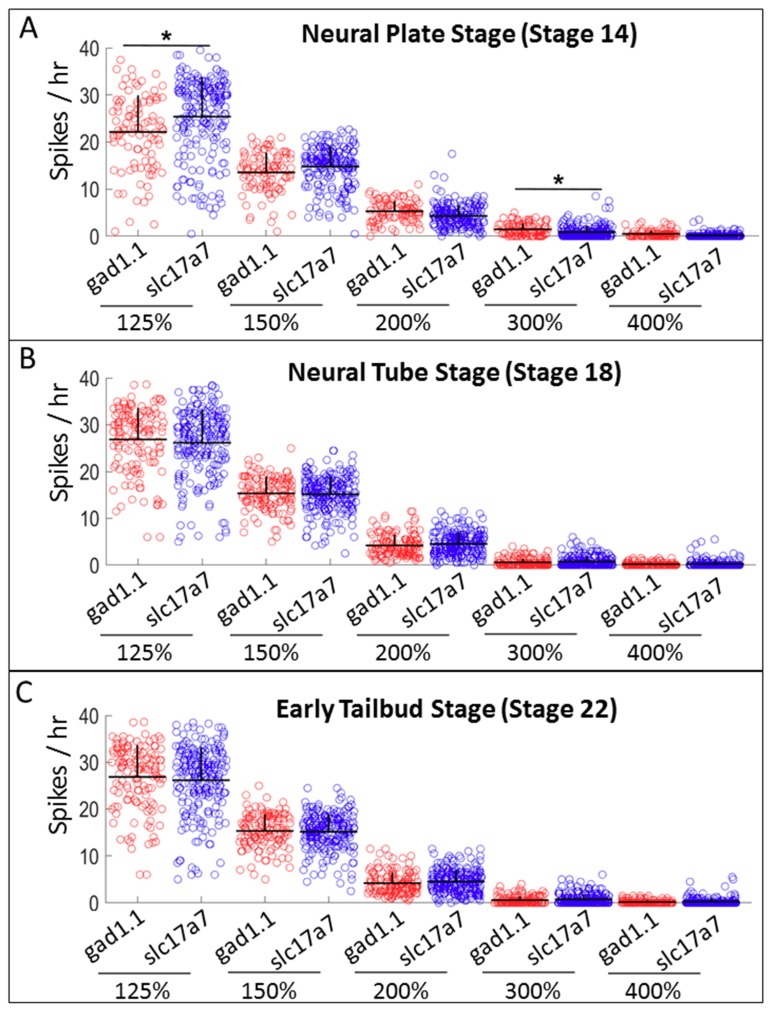
Presumptive glutamatergic cells dissected at neural plate stage of development show more low-level spiking activity, while presumptive GABAergic cells display higher-amplitude spiking. Comparison of spiking frequency counted using five different thresholds (125%, 150%, 200%, 300% and 400% of baseline), between inhibitory (*gad1.1*) and excitatory (*slc17a7*) neurons at: (**A**) neural plate stage (Stage 14); (**B**) neural tube stage (Stage 18); and (**C**) early tailbud stage (Stage 22). Stars represent statistically significant differences according to both Bonferroni-corrected two-sample Kolmogorov–Smirnov Test (*p* < 0.05) and Cohen’s d statistics for effect size (mean + SD; *n* = 5 cultures; * 0.2 ≤ |d| < 0.5).

Using spike counting to characterize calcium activity, we once again observed significant differences at the neural plate stage. Interestingly, the neurotransmitter phenotype that was associated with significantly higher spiking activity was dependent upon the particular threshold selected. At the neural plate stage (Stage 14), *slc17a7*-positive cells exhibit a higher number of spikes when the threshold for spike determination was set at 125% of baseline, whereas *gad1.1*-positive cells exhibited significantly higher spiking activity when the threshold for identifying a spike was set at 300% of baseline (Figure 2A and Figure S1D). That is to say, we observed a switch in the phenotype associated with higher spiking based on the particular threshold value selected to classify spiking behavior.

**Figure 3:**
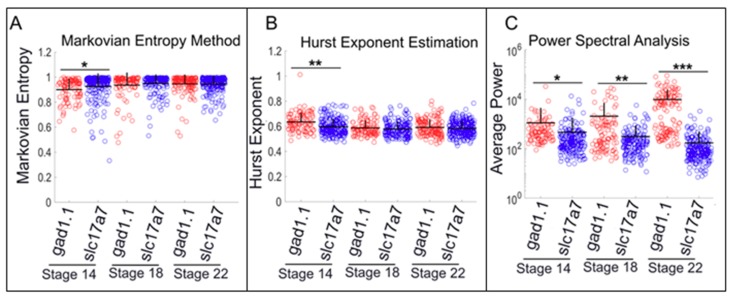
Presumptive glutamatergic cells dissociated at neural plate stage show more chaotic activity, while presumptive GABAergic cells display more periodic calcium dynamics. Comparison of Markovian entropy, estimated Hurst exponent, and average power between inhibitory (*gad1.1*) versus excitatory (*slc17a7*) neurons at: (**A**) neural plate stage (Stage 14); (**B**) neural tube stage (Stage 18); and (**C**) early tailbud stage (Stage 22). Stars represent statistically significant differences according to both Bonferroni-corrected two-sample Kolmogorov–Smirnov Test (*p* < 0.05) and Cohen’s d statistics for effect size (mean + SD; *n* = 5 cultures; * 0.2 ≤ |d| < 0.5, ** 0.5 ≤ |d| < 0.8, *** |d| ≥ 0.8). Markovian entropy was calculated with *n* = 4 and *k* = 1.

These results are corroborated by the other metrics we employed to assay calcium activity. *Gad1.1*-positive cells exhibited higher average power across all the stages compared to *slc17a7*-positive cells, suggesting that the former population is characterized by calcium activity patterns with greater periodicity (Figure 3C and Table S4). This was the case at later stages (Stage 22) even when a higher threshold for FISH signal was used to classify positive cells (300% and 400% of the background) (Figures S15 and S17). It is also consistent with the spike-counting results that *gad1.1*-positive cells exhibited higher estimated Hurst exponent (representing more persistency in the calcium signal) and lower entropy (more predictability) when compared to *slc17a7*-positive cells. However, statistical significance was limited to the early neural plate stages (Figure 3A,B).

Taken together, these observations suggest that inhibitory and excitatory neurons at neural plate stages do show, although with moderate effect size (as measured by Cohen’s d), and not at all stages, some statistically significant difference in the patterns of calcium activity correlated with these different neurotransmitter phenotypes. Presumptive glutamatergic cells show more low-amplitude spiking and more chaotic low-level calcium activity, while presumptive GABAergic cells display higher-amplitude spikes with more periodic behavior during specific stages of development.

### 2.5. The Intensity of Gene Expression for sox2 and tubb2b is not Directly Correlated with the Level of Calcium Activity

Given that we observed modest correlations between calcium activity and neurotransmitter markers, we then tested whether calcium activity correlated with an earlier, more fundamental decision in neural development in which one population off cells becomes committed to differentiation (*tubb2b*-expressing cells) while a second population remains in an undifferentiated progenitor state (*sox2*-expressing cells). Analogous to the analysis performed with *slc17a7* and *gad1.1*, we asked whether the intensity level of gene expression for *sox2* or *tubb2b* correlates with the levels of calcium activity in either a linear or nonlinear fashion. Following calcium imaging, we therefore analyzed our dataset using spike counting and average power, Hurst exponent estimation and Markovian entropy measurements. We correlated cellular calcium dynamics revealed by each of these measures with the levels of expression of the marker genes quantified as a FISH intensity score by performing both a linear and a nonlinear correlation analysis using Pearson correlation coefficient and R-squared value of general additive model between FISH score and calcium dynamics. No significant correlation was seen between calcium activity and the intensity of fluorescence signal for either *sox2* or *tubb2b* by any of the metrics we used (Tables S1 and S2). As baseline information for marker gene expression, we also performed a Kruskal–Wallis test to determine whether the percentages of cells expressing either sox2 *or tubb2b* at the various FISH levels are similar across the three developmental stages. The percentage of cells displaying both *sox2* and *tubb2a* expression gradually increases from neural plate to neural tube stages, and begins to decrease by tailbud stages as the state of neural determination becomes increasingly fixed.

### 2.6. Cells that are Positive for tubb2b or sox2 Show Different Patterns of Calcium Activity than Cells that are Negative for these Markers

Although the intensity of gene expression did not correlate with the levels of calcium activity, we wished to test whether calcium activity was significantly different in cells that were positive for either *sox2* or *tubb2b* versus those that were negative for the same marker. Using spike counting to assess calcium activity, *tubb2b*-positive cells were shown to exhibit a lower number of spikes than *tubb2b*-negative cells at a spike threshold of 125% of baseline, but a higher number of longer-duration spikes reaching 200%, 300%, and 400% of baseline at both neural plate and early tailbud stages (Figure S2 and Table S5). Similar results were observed at a higher FISH score cut off values, but only at Stage 14 (Figures S4 and S6). Interestingly, neural progenitor cells at the neural tube stage (Stage 18) showed a higher number of spikes when a threshold value of 200% of baseline was applied (Figures S10 and S12) suggesting that this stage represents a transitional point in cellular state.

While other measures of calcium activity present a slightly more complicated picture of calcium activity, they are generally consistent with the spike-counting data. *Tubb2b*-negative cells show higher entropy than *tubb2b*-positive cells at Stages 14 and 22, while *sox2*-positive cells are characterized by higher entropy values at neural tube stages (Stage 18) (Figures S2 and S14). *Sox2*-positive progenitor cells exhibit higher power (periodicity) as compared to *sox2*-negative cells at Stages 14 and 18 at all FISH score cutoff values, and *tubb2b*-negative cells exhibit higher power value at the early tailbud stages (Stage 22) (Figures S2, S4, S6, S8, S10, S12, S14, S16 and S18).

Taken together, our results suggest that there are significant differences in calcium activity between cells that are positive for *tubb2b* versus those that are negative for *tubb2b*, and likewise between those that are positive and negative for *sox2*. This effect is most prominent at neural plate stages, at which *tubb2*-negative and *sox2*-positive cells display higher levels of low-amplitude spiking, while *sox2*-negative and *tubb2*-positive cells display more high-amplitude spiking activity.

### 2.7. Neural Progenitors and Differentiated Cells Exhibit Significantly Different Patterns of Calcium Activity

**Figure 4:**
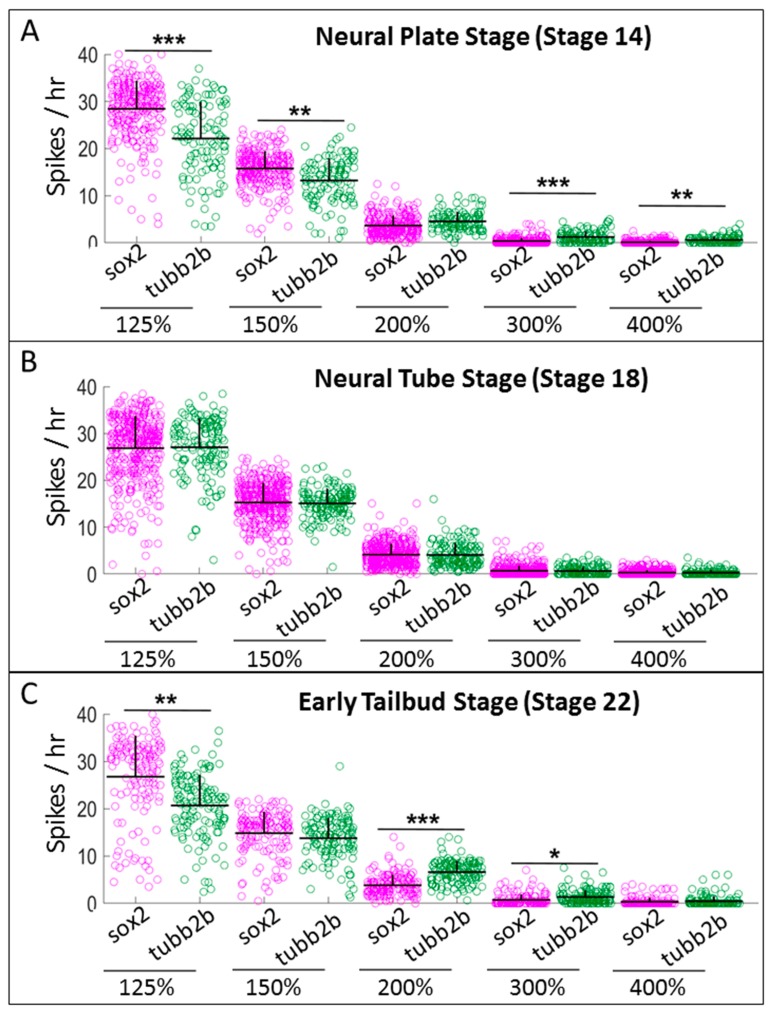
Neural progenitor cells exhibit high numbers of small spikes and lower numbers of high-amplitude spikes, but differentiated neurons exhibit a lower number of small spikes and larger number of high-amplitude spikes. Comparison of spiking frequency counted using five different thresholds (125%, 150%, 200%, 300% and 400% of baseline), between neural progenitor cells (*sox2*) and differentiated neurons (tubb2b) at: (**A**) neural plate stage (Stage 14); (**B**) neural tube stage (Stage 18); and (**C**) early tailbud stage (Stage 22). Stars represent statistically significant differences according to both Bonferroni-corrected two-sample Kolmogorov–Smirnov Test (*p* < 0.05) and Cohen’s d statistics for effect size (mean + SD; *n* = 5 cultures; * 0.2 ≤ |d| < 0.5, ** 0.5 ≤ |d| < 0.8, *** |d| ≥ 0.8).

To determine whether *sox2*-positive progenitor cells displayed significantly different patterns of calcium activity when compared with cells committed to differentiation, we compared patterns of calcium activity in *sox2*-positive cells versus *sox2*-negative cells as well as in *tubb2b*-positive versus *tubb2b*-negative cells. As described previously, we used 200% of the background signal as the threshold for a positive cell, although to avoid a single arbitrary threshold, several higher thresholds were also employed in our analysis. Using spike counting to characterize calcium activity, we once again observed significant and robust differences at the neural plate and early tailbud stages. *Sox2*-positive neural progenitors consistently exhibit higher number of spikes at lower amplitudes (threshold set at 125% and 150% of baseline activity), while *tubb2b* cells show higher levels of spiking at high amplitudes (thresholds of 200–500% of baseline) (Figure 4A,C)

**Figure 5:**
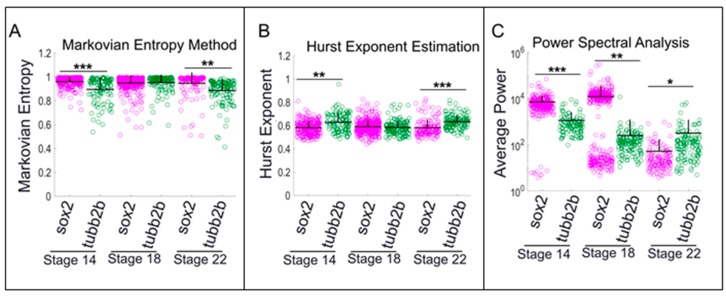
Neural progenitor cells exhibit comparatively noisy low-level calcium dynamics, while differentiated neurons exhibit relatively persistent and more predictable calcium dynamics. Comparison of Markovian entropy, estimated Hurst exponent, and average power between neural progenitor (*sox2*) versus differentiated neurons (tubb2b) at: (**A**) neural plate stage (Stage 14); (**B**) neural tube stage (Stage 18); and (**C**) early tailbud stage (Stage 22). Stars represent statistically significant differences according to both Bonferroni-corrected two-sample Kolmogorov–Smirnov Test (*p* < 0.05) and Cohen’s d statistics for effect size (mean + SD; *n* = 5 cultures; * 0.2 ≤ |d| < 0.5, ** 0.5 ≤ |d| < 0.8, *** |d| ≥ 0.8). Markovian entropy was calculated with *n* = 4 and *k* = 1.

Other measures of calcium activity were consistent with the spike counting results. *Sox2*-positive progenitor cells exhibited lower Hurst exponent and higher Markovian entropy measures compared to differentiated cells at all stages (Figure 5 and Figures S2, S4, S6, S8, S10, S12, S14, S16 and S18). Additionally, *sox2*-positive cells exhibit higher average power (more periodicity) at Stages 14 and 18; by the late neurula stages, *tubb2b*-positive cells show higher average power (Figure 5). Interestingly, at neural tube stages, some cells exhibit low average power while others exhibit high power, generating a bimodal distribution and suggesting this to be a transition stage during which *sox2*-positive cells begin to express *tubb2b* and, accordingly, exhibit stronger periodicity (Figure 5C). Overall, these analyses suggest that neural progenitor cells exhibit comparatively noisy low-amplitude calcium dynamics, particularly at neural plate stages. This activity is characterized by a high number of small spikes and lower number of high-amplitude spikes. Conversely, differentiated neurons exhibit relatively persistent and more predictable calcium dynamics characterized by fewer small spikes and more high-amplitude spikes of longer duration.

## 3. Discussion

The overarching goal of this study was to determine if calcium activity was unambiguously correlated with specific genetic phenotypes on a single-cell level, rather than only correlated on a population level. We focused our study on three specific questions. First, we asked if, at a single-cell level, there is a correlation of neurotransmitter phenotype (*slc17a7* and *gad1.1*) with specific patterns of calcium activity. We also queried if there was any correlation between calcium activity and neural stem cells (*sox2*) or cells committed to differentiation (*tubb2b*). Secondly, we asked if calcium activity (and its association with gene expression) requires continuous cell–cell interactions. To this end, we assessed whether expected calcium activity patterns persisted in cells isolated from their neighbors. Finally, given that calcium activity in the early embryonic nervous system does not display the stereotypical characteristics of calcium activity in mature neurons, we asked if using different methods of analysis of calcium activity led to different conclusions, which has important implications for the interpretation of calcium activity data.

A substantial body of work has suggested that specific patterns of calcium spiking behavior lead to the adoption of specific neurotransmitter phenotypes, with elevated levels of calcium transients leading to an increased tendency towards acquisition of inhibitory neurotransmitter phenotypes and lower levels of spiking activity associated with the acquisition of excitatory phenotypes. This type of activity has even been shown to mediate subsequent re-specification of neurotransmitter phenotype in the mature nervous system [25]. While the evidence demonstrating this mechanism of phenotype determination is compelling, many of these studies were performed on a population level rather than attempting to correlate calcium activity in single cells with a given phenotype. The importance of understanding calcium dynamics at the single-cell level has been established in recent work [43].

While we did not find the level of expression of any of our genes of interest to scale or correlate with the level of calcium activity, there were statistically significant differences between *gad1.1*-positive cells and *gad1.1*-negative cells, as well as between *slc17a7*-positive and *gad1.1*-positive cells at neural plate stages. *Gad1.1*-negative cells showed significantly more spiking than *gad1.1*-positive cells when lower spike definition thresholds were applied. However, applying higher spike definition thresholds (200% and 300% of baseline calcium activity) led to the conclusion that *gad1.1*-positive cells showed significantly more spiking activity than *gad1.1*-negative cells. Consistently, *slc17a7*-positive cells exhibit higher levels of low-amplitude spiking than *gad1.1*-positive cells, whereas *gad1.1*-positive cells exhibit higher levels of higher-amplitude spiking than *slc17a7*-positive cells (Figure S1). This low-level spiking that characterizes glutamatergic cells has not previously been reported or associated with neurotransmitter phenotype; however, it remains unclear what, if any, physiological relevance can be attributed to this activity pattern.

Given the importance of neurula stages for the fundamental cellular decision between differentiation and remaining a progenitor cell, we also addressed whether calcium activity was significantly different between these two basic cell types (as defined by the expression of *sox2* for neural progenitor cells and of *tubb2b* for cells committed to differentiation), a comparison not previously well-studied. Surprisingly, there were robust and significant differences between the two categories of cells, with *sox2*-positive cells displaying low-amplitude, high-frequency spiking and *tubb2b*-positive cells committed to cell cycle exit and differentiation showing increased high-amplitude spiking. There are relatively few studies that associate patterns of calcium activity with this important cellular decision. One study [41] failed to observe any calcium activity in cells dissected from the spinal cord at tailbud stages. However, this discrepancy can be attributed to stage differences or, perhaps more likely, to the application of a single spike definition threshold too high to detect the significant amount of low-amplitude, high-frequency spiking we observe in our report. Some papers report that SOX2 reduces calcium influx and activity from the endoplasmic reticulum, while other reports show that blocking internal calcium stores decreases cellular proliferation and self-renewal [44,45]. Such apparent discrepancies could reflect the application of different thresholds for classifying calcium activity patterns. It would be useful to determine if the clear differences in calcium activity between progenitors and differentiating cells that we observe in our study are present in vivo, as well as to investigate whether inducing frequent low-amplitude spiking can promote or maintain a neural progenitor state in cells that would otherwise undergo differentiation.

This study also addressed the degree to which isolated primary cells in culture demonstrate cell-autonomous calcium activity. To do so, we dissected neural plates or neural tubes, dissociated the tissues, and recorded calcium activity in single cells in the absence of endogenous cell–cell communication. We found that the calcium activity patterns and phenotype profiles were distinctive for each of the three developmental stages analyzed. At the neural plate stages, there were robust differences between progenitor cells and those cells specified to undergo differentiation, as well as modest differences between presumptive GABAergic and glutamatergic cells. By neural tube stages, this profile changed, and few differences in calcium activity could be detected between cells expressing the different phenotypes. By early tailbud stages, we found no significant differences in calcium activity between glutamatergic and GABAergic cells and, although differences were still detected between progenitor cells and those committed to differentiation, they were less robust than at earlier developmental stages. Given the role of calcium activity in mechanical stress and response to perturbation, it remains possible that the procedure of plating cells inherent in primary cell culture intrinsically alters calcium activity. Nevertheless, we found clear differences in calcium activity between developmental stages, suggesting that the primary cell culture is faithfully reflecting the given phenotypic state of the cells. Determining whether these differences reflect in vivo conditions will require further experimentation using imaging of precisely tracked cells.

A final goal of this study was to apply multiple methods of analysis to calcium activity to obtain a more complete understanding of the underlying patterns. Given the non-canonical and irregular nature of calcium activity in the early, embryonic nervous system as well as the lack of a consistent definition of what constitutes a spike in the current literature, we theorized that the application of different analysis methods and threshold parameters could dramatically affect the conclusions drawn from a dataset. Different groups have defined a spike as: (i) an instance of fluorescence intensity greater than 150% of the baseline (dF/F0) [34]; (ii) a transient amplitude with a rise time of 5 s reaching more than 20% of the baseline value (dF/F0) as determined by the values over the previous 10 min [39]; (iii) a transient amplitude with a rise time of 5 s that exceeds twice the baseline variation during the previous 10 min [46]; and (iv) any transient lasting less than 3 min and with an amplitude of more than three standard deviations above the average value (>3 SD) [35]. Furthermore, the method of baseline establishment differs between even between these reports, complicating any attempt to synthesize conclusions from the current body of published literature.

In light of this, we did not select a single threshold for defining a spike, but instead chose to analyze our data using multiple thresholds for spike identification. Analyzing our data at different thresholds revealed several novel correlations/associations, a finding which in and of itself highlights the effect that researcher choice can play in influencing experimental conclusions. If the threshold is set to 125% or 200% of the baseline (i.e., when low-amplitude spikes were counted), presumptive glutamatergic cells were correlated with higher levels of calcium activity, while at higher thresholds, GABAergic cells were correlated with significantly higher levels of calcium activity. Likewise, at both neural plate and tailbud stages, when the threshold was set to 125% or 200% of the baseline, progenitor cells were correlated with higher levels of calcium activity. Conversely, at higher amplitude thresholds, cells specified to differentiate (i.e., *tubb2b*-positive cells) were correlated with significantly higher levels of calcium activity. Using a wide range of thresholds was the only way to determine that both cell types were statistically significantly correlated with distinct patterns of spiking activity that would have been obscured by analysis at a single threshold. We believe that this kind of approach will be essential for deciphering the cellular code of calcium activity, particularly during early stages of development, during which activity patterns are much less stereotypical that the activity seen in mature neurons.

There have been several attempts to employ more comprehensive methods to probe calcium activity by extending analysis beyond simply counting spike frequency. These include Bayesian inference [47], supervised learning techniques [48,49], and gating models of RyR [50]. These approaches incorporate additional characteristics of spikes, such as time of spike initiation, rise and termination, peak fluorescence during spiking, and spike spanning duration [48]. These methods work extremely well for mature neurons and cardiac myocytes, cell types that have a predicted and stereotypical shape of calcium spikes and known noise statistics, neither of which holds true in embryological systems. Given the irregular patterns of calcium activity in early neural development, it is also often beneficial to employ analytical approaches that go beyond spike counting to discern more global patterns of calcium activity. Limiting analytical methods to spike counting can exclude much of the more complex dynamics underlying calcium patterns. To overcome this problem, we employed power spectral analysis, fractal analysis, and entropy analysis to quantify the complexity of a signal in a more holistic and comprehensive way.

Calculating average power can reveal the periodicity of a signal that arises from a combination of multiple factors [37,51,52]. In our study, this metric suggested that there is less periodicity in calcium signal dynamics recorded from neural progenitors and inhibitory neurons as compared to cells committed to differentiate into neurons and excitatory neurons, respectively. As high calcium activity has been implicated in proliferation, differentiation of neural progenitors to neurons [53,54], morphogenetic movements, and neural tube closure [55] at these stages of development, the high average power measured in our neural progenitor cells at Stages 14 and 18 could be due to a higher number of small amplitude spikes that span shorter duration during neural tube closure. Although we do not know the underlying causes of variation that leads to our observed bimodal distribution of calculated average power of neural progenitor cells at Stage 18 (Figure 5C), our speculation is that this could be due to a transitional stage; at neural plate stages only a tiny fraction of these cells show low power, while at neural tube stages it is more even and at tailbud stages virtually all progenitor cells have low average power. This could reflect the transition from a highly active state of cells during morphogenetic movement and neural tube closure to an early tailbud stage with cells committed to differentiation.

The Hurst exponent provides a measure for long-term memory or self-similarity of a time series. Our results show that the average of the estimated Hurst exponent of each cell type was above 0.5, indicating a time series with a positive autocorrelation and a persistent type of calcium dynamics. However, this metric also showed that *gad1.1-* and *tubb2b*-positive cells had significantly larger estimated Hurst exponents than *sox2*- and *slc17a7*-positive cells, suggesting that presumptive GABAergic cells and neurons committed to a differentiation pathway exhibit persistent calcium transients with high degrees of self-similarity. Markovian entropy provides a measure of the predictability of the time series, regardless of whether it is characterized by periodic or more irregular features [40,56,57]. Measured between 0 and 1, a low value indicates a predictable signal, whereas a value closer to 1 signifies an unpredictable signal. Our results suggest that *sox2*-positive cells and *slc17a7*-positive cells exhibit higher Markovian entropy than *tubb2b*- and *gad1.1*-positive cells, suggesting that neural progenitors and excitatory cells exhibit a less predictable signal characterized by a high number of small-amplitude spikes that span shorter durations, whereas inhibitory neurons and differentiated neurons exhibit a comparatively predictable signal characterized by a high number of high-amplitude spikes of longer duration. These results are consistent with the results of our Hurst exponent analysis.

## 4. Materials and Methods

### 4.1. Cell Culture and Calcium Imaging

A schematic overview of our procedures is provided in Figure 1. All animal care and use procedures were performed in accordance with policies of the Institutional Animal Care and Use Committee (IACUC, approved 11-21-16, #2016-11-21-11567-mssaha) at the College of William & Mary following standard methodologies [42]. To obtain embryos, *Xenopus laevis* females and males received 600 U/mL and 400 U/mL, respectively, of human chorionic gonadotropin (Chorulon, Madison, NJ, USA) to induce embryo production. Embryos were collected and de-jellied in a 2% cysteine solution (pH ~8.0) and raised until either neural plate (Stage 14), neural tube (Stage 18) or early tailbud stages (Stage 22). These particular stages were selected for two reasons. First, they represent stages with clear morphological landmarks enabling consistency and accuracy in staging. Secondly, these stages represent key developmental landmark stages: Stage 14 represents the onset of neurulation; Stage 18 displays the unambiguous neural tube stage; and Stage 22 represents the beginning of tailbud elongation. Staging was carried out according to Nieuwkoop and Faber (1994) [58]. For each of the three stages, four pieces of the presumptive neural ectoderm of embryos were dissected in 0.1X Marc’s Modified Ringer solution (MMR: 10 mM NaCl, 0.2 mM KCl, 0.1 mM MgSO_4_, 0.5 mM HEPES, 0.01 mM EDTA, and 0.2 mM CaCl_2_, pH adjusted to 7.4–7.6) supplemented with 1 mg/mL collagenase B (Roche, Mannheim, Germany) to aid the dissection process. A minimum of five embryos for each stage were used for analysis. The dissected explants were incubated at room temperature in calcium- and magnesium-free (CMF) solution (116 mM NaCl, 0.67 mM KCl, 4.6 mM Tris, and 0.4 mM EDTA; pH 7.8) [34] for 1 h to dissociate the cells. Following tissue dissociation, the cells were plated on 35 mm Nunclon dishes (Cellattice; Nexcelom, Lawrence, MA, USA) in culture solution containing 2mM Ca^2+^ (116 mM NaCl, 0.67 mM KCl, 2 mM CaCl2, 1.31 mM MgSO4, and 4.6 mM Tris; pH 7.4) [29] for 1 h and were then treated with 2.5 μM Fluo4-AM/0.01% Pluronic F-127 acid for an additional hour. Cells were imaged using either a Zeiss LSM 510 or Nikon A1R T*i* confocal laser scanning microscope after three successive washes in culture solution. Calcium activity was recorded for 2 h at 0.125Hz using Argon 488 nm laser. Following imaging, cells were fixed in 1X MEMFA (100 mM MOPS (pH 7.4), 2 mM EGTA, 1 mM MgSO_4_, 3.7% (*v/v*) formaldehyde) for 1 h and stored in 1× Phosphate Buffered Saline Solution (PBS) at 4 °C (Figure 1) for further analysis. The raw data for calcium imaging are available in Table S6.

### 4.2. Calcium Imaging Analysis

Overview: Given that even cultured cells move during imaging, we used the “Object Tracking” function of Nikon NIS-Elements to identify individual cells and their associated calcium activity. Bright-field images were used to assign each cell a unique region of interest (ROI) using the “spot detection” tool. To ensure that every cell Was detected appropriately as an individual ROI, circularity and contrast settings were adjusted to include the maximal number of cells. To be included in the dataset, a cell had to be detected in a minimum of 600 contiguous time frames (45 min of imaging). Accuracy of ROI assignment and tracking was confirmed manually through visual inspection for each experiment. ROIs that did not perfectly correspond to single cell were manually deleted. Fluorescent intensity of individual cells (as measured using the FITC channel) was then exported as a .csv file. As fluorescent signals are often observed to display overall upward or downward trends during imaging, we employed a Baseline Correction de-trending algorithm developed by Eilers and Boelen [40,59]. To obtain a more comprehensive view of non-canonical calcium activity characteristic of early embryogenesis, we performed four different types of analysis: determination of spiking frequency, the average power, Hurst exponent and Markovian entropy [40].

Spike counting: The vast majority of studies that analyze calcium activity do so by counting spike frequency, defined as the number of times the level of fluorescence emitted by a labeled calcium indicator rises above a certain threshold. However, both the precise method of identifying spikes and the amplitude threshold used as a cutoff are inconsistent in the literature. Here, we defined a spike as an increase in fluorescent signal that spans at least two time frames with a threshold intensity value above baseline (defined as the average value of all time points). To be as unbiased as possible, we applied multiple thresholds, namely 125%, 150%, 200%, 300%, 400%, and 800% of baseline, in our determination of spikes per hour per cell.

Average power calculation: We also examined the average power for each time series, which is a nonparametric measure that roughly assesses the extent to which a time-varying signal is periodic. In this study, we calculated the average power for our time series of calcium activity using the following formula, where *P* denotes average power, *X_i_* denotes data points over the time domain and *T* denotes the total number of time frames.

(1) $$P=\;\frac{1}{T}\overset{T}{\underset{i=1}{\sum }}X_{i}^{2}$$

Estimation of Hurst Exponent: Hurst exponent analysis determines the self-similarity in a time series by measuring the decrease in autocorrelation as a function of an increase in the time lag. For our data, we calculated the Hurst Exponent with a Rescaled Range (R/S) [60]. This procedure estimates the Hurst Exponent as the slope of the regression line between averaged rescaled range value and the logarithm (base 2) of the time window length [40]. The Hurst Exponent (H) should range between 0 and 1, with 0.5 < H < 1 indicating persistent dynamics in the time series—in other words, future time points are more likely to follow the trend observed at a given time point. Conversely, 0 < H < 0.5 indicates anti-persistent dynamics, that is, future time points are more likely to reverse the trend observed at a given time point. For time series with autocorrelation close to 0, such as a Gaussian white noise signal, H should be close to 0.5.

Markovian entropy measurement: We also calculated the Markovian entropy of each time series, which measures the predictability of the observed dynamics by treating the observed signal as the realization of a Markov Process [40]. Calculating the Markovian Entropy implies two assumptions: first, the fluorescent intensity obtained from calcium imaging of a single cell can transition between *n* discrete states with a defined probability, and, second, only the last *k* states affect the conditional probability for future behavior of that particular cell (memoryless property).

(2) $$E=-\overset{n}{\underset{i=1}{\sum }}P_{i}log_{2}(P_{i})$$

We discretized our dataset and generated *nk* by *n* transition probability matrix setting *k* = 1 and *n* = 4 quantiles which is consistent with suggested settings as described in [40]. We then calculated the Shannon entropy E for each row of the transition probability matrix using Equation (2), where $P_{i}$ represents the value in the *i*-th column of a given row. We then summed the resulting entropy values from each row, and normalized the sum by $n^{k}\log_{2}\left(n\right)$ to obtain the Markovian Entropy. The Markovian Entropy ranges from 0 to 1, where a larger entropy value implies less predictable dynamics in the time series. Taken together, these methods provide a more comprehensive analysis of calcium activity that reduces a priori arbitrary thresholds or a posteriori biases to fit data. When discussing each of the techniques individually, the specific name of that method is used. However, when referring to the collective output of several methods of analysis, we employ the term “metrics of calcium activity”.

### 4.3. Fluorescence In situ Hybridization (FISH)

Antisense mRNA probes complementary to endogenous mRNA were synthesized to perform in situ hybridization on cultured cells to determine their molecular phenotype. Excitatory and inhibitory phenotypes were assayed using markers for the vesicular glutamate transporter 1 *vGlut1*, now known as solute carrier family 17 member 7, *slc17a7* (NM_001089635.1) [61], and glutamic acid decarboxylase 1, *gad1.1* (NM_001085801) [62] respectively. The state of neuronal determination was assayed by using a probe for Sry-related HMG factor *sox2* (AF022928.1) [63] to identify neural progenitor cells, while cells committed to differentiation were identified by the expression of neural beta-tubulin *tubb2b* (BC044030) [64]. Fluorescent in situ hybridization was conducted in accordance with the protocols from David and Keller [65] using an anti-digoxigenin peroxidase antibody (Roche) and fluorescein–tyramide (GE Healthcare, Buckinghamshire, England) as the color substrate. Images were captured with Nikon A1R T*i* or Zeiss LSM 510 confocal microscope after completion of FISH assay (Figure 1). All FISH data are available upon request.

### 4.4. Calculation of FISH Score

The intensity of the fluorescent signal in the FISH assays were obtained by importing still images into Nikon NIS-Elements Software and applying built-in “spot detection” and “measurement” functions (as described in Calcium Image Analysis). The ROI IDs and the corresponding measurements, including “Mean Intensity” fluorescent values of individual cells, were exported as .csv files for further analysis. The last frame of the calcium image and the in situ image were co-registered using GNU Image Manipulation Program (GIMP 2.8.16) to match calcium ROI ID and FISH ROI ID of each cell. The fluorescent intensity (F) of every cell was normalized with an average of the lowest five values (F0, or background) and each cell was assigned its own FISH score (F/F0). Therefore, the FISH score is a dimensionless quantity that quantifies the relative levels of expression of marker genes. We analyzed the correlation between phenotype and calcium activity both with and without arbitrary FISH score thresholds. For the former analysis, we considered a cell positive for a given signal if its value was 200% or more of the average of the five cells with the lowest fluorescence on the same plate. For the latter analysis, we performed correlation analysis between the normalized FISH score and the metrics for calcium activity described above.

### 4.5. Statistical Analysis

We calculated Pearson’s correlation coefficient and the nonlinear association coefficient R^2^ value of the general additive model using the GAM package in R [66,67] to understand the relationship between levels of expression of marker genes and cellular calcium activity. We also calculated an effect size measure, Cohen’s d value, to gain insight into the magnitude of the difference between defined cell populations. To discern statistically different distributions of measures (spike count, spike duration, average power, Hurst exponent, and Markovian entropy) between two cell populations of interest, we used the Bonferroni-corrected two-sample Kolmogorov–Smirnov (K-S) Test. The calcium dynamics were considered to be significantly different between two populations only if *p* < 0.05 and |Cohen’s d| > 0.2. To validate the results of the Hurst exponent and Markovian entropy analyses, we performed randomization controls. The sequential order of data points is essential if a long-range memory exists (persistency) in a signal. Given that Hurst exponent and Markovian entropy quantify this type of inherent sequential order of a signal, data shuffling destroys structural composition and results in a Markovian entropy measure close to 1 and a Hurst exponent close to 0.5. To validate the significance of our results, we randomized our dataset by taking the same number of values from the original dataset at random using a MATLAB built-in function, “randsample,” and compared our results against a random sampling. We observed an estimated Hurst exponent of 0.47 ± 0.023 and Markovian measure of 0.996 ± 0.002 in our randomized datasets, as expected. To validate gene expression analyses, we also randomized our gene expression data by combining all cells from two experiments (e.g., *tubb2b* and *sox2*), sorting them based on FISH score, and then dividing them into two groups. No statistically significant difference was ever detected in these randomized groups.

## 5. Conclusions

In this study, we addressed three major questions. First, on a single-cell level, we asked if there is a correlation of phenotype with calcium activity for several critical neuronal phenotypes. While the intensity level of gene expression did not correlate with level of calcium activity in a linear or nonlinear manner, when using thresholds for gene expression and calcium activity, we did find statistically significant correlations between calcium activity patterns and cells displaying specific phenotypes (as assayed by gene expression). We showed that high-frequency spiking activity is correlated, on the single-cell level, with excitatory, glutamatergic cells at low-amplitude thresholds, but with inhibitory, GABAergic cells at high-amplitude thresholds. We also queried whether there was any correlation between calcium activity and the state of cellular differentiation (with neural precursors characterized by *sox2* expression, and differentiating cells characterized by *tubb2b* expression). Surprisingly, we also found that high-frequency spiking activity is correlated with progenitor cells at low amplitudes and with cells committed to differentiation at higher amplitudes. Furthermore, measures of Markovian entropy, Hurst exponent, and power spectral analysis suggested that newly differentiated neurons exhibit relatively persistent and predictable calcium activity compared to the more irregular activity of neural progenitor cells.

Secondly, we asked if calcium activity requires continuous cell–cell interactions, or if cells isolated from neighbors still display unique patterns of calcium activity. Our study showed that cells at each of our assayed stages did indeed display a characteristic pattern of calcium activity, although more precise imaging would be required to confirm the degree to which the patterns we observed in vitro match those present in vivo. Finally, given that calcium activity in the developing nervous system does not display stereotypical activity patterns characteristic of mature neurons, we asked if using different methods of analysis of calcium activity could lead to a more comprehensive portrait of the patterns specific to each cell type. With respect to spiking, given that presumptive excitatory glutamatergic cells as well as progenitor cells are correlated with higher frequency spiking at lower amplitudes and differentiating and GABAergic cells are correlated with elevated spiking frequency at higher thresholds, our study showed that using multiple thresholds for defining a spike is essential for a full understanding patterns of calcium activity. Finally, our study demonstrated that multiple methods of data analysis that capture the global features of the signal (such as periodicity, predictability and autocorrelation) provide a more complete picture of the complex nature of calcium activity that characterizes developing systems. Understanding and analyzing this activity is essential, given that small perturbations that deregulate this activity can have long-term consequences for the health of the organism.

## Supplementary Materials

Supplementary materials can be found at https://www.mdpi.com/1422-0067/20/8/1880/s1.
