# Supplementary material for: Calcium Activity Dynamics Correlate with Neuronal Phenotype at a Single Cell Level and in a Threshold-Dependent Manner
Source: Int J Mol Sci. 2019 Apr 16;20(8):1880. doi: 10.3390/ijms20081880 (PMC6515432; doi:10.3390/ijms20081880)
Supplement: Supplementary file 1 [file ijms-20-01880-s001.zip › Supppl_Data_pdf_figs/Supplementary figures with figure legend.pdf]

International Journal of *Molecular Sciences*

MDPI

Calcium Activity Dynamics Correlate with Neuronal Phenotype at a Single Cell Level and in a Threshold-Dependent Manner

Sudip Paudel#, Eileen Ablondi#1, Morgan Sehdev[2], John Marken[3] Andrew Halleran[3], Atiqur Rahman[4], Peter Kemper[4] and Margaret Saha

Department of Biology, College of William and Mary, Williamsburg VA

1. Current address: Harvard University, Boston MA
2. Current address: Harvard University School of Medicine, Boston MA
3. Current address: California Institute of Technology, Department of Bioengineering, Pasadena CA
4. Department of Computer Science, College of William and Mary, Williamsburg VA

#These authors contributed equally.

Supplementary Table 1: Pearson correlation coefficients between calcium measures and FISH scores.

Supplementary Table 2: GAM R squared coefficients between calcium measures and FISH scores.

Supplementary Table 3: Cohen's d and K-S p value at different FISH score threshold between marker positive and marker negative cells.

Supplementary Table 4: Cohen's d and K-S p value at different FISH score threshold between inhibitory (*gad1.1*) and excitatory (*slc17a7*) neurons.

Supplementary Table 5: Cohen's d and K-S p value at different FISH score threshold between neural progenitors (*sox2*) and differentiated neurons (*tubb2b*).

Supplementary Table 6: De-trended calcium imaging data. The first column represents the cell ID from 60 experimental conditions (3 stages, 4 genes, and 5 replicates), and columns 2 to end is time series fluorescent intensity. FISH data and manual ID registration data is available upon request.

International Journal of
Molecular Sciences

MDPI

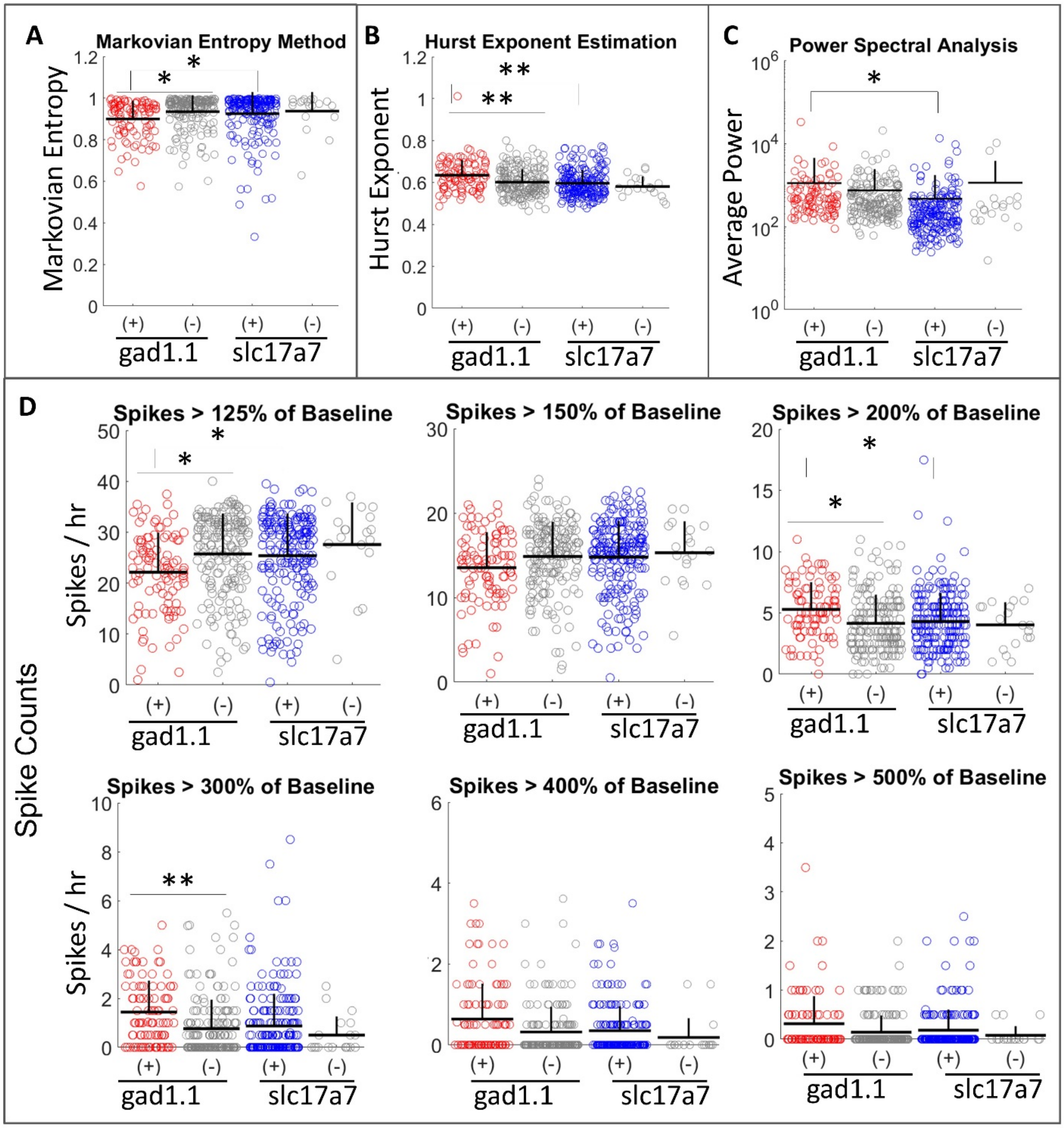

Supplementary Figure 1: Comparison of calcium activity between *gad1.1*-positive (inhibitory) neurons, *gad1.1* negative cells, *slc117*-positive (excitatory) and *slc17a7* negative cells (cells with a FISH signal intensity 200% or more than five lowest cells on the same experimental plate were considered positive for expression of the gene of interest). Calcium activity was quantified using (A) Markovian Entropy Measure (B) Hurst Exponent (C) Average power (D) Spike frequency counted at different thresholds (125%, 150%, 200%, 300%, 400%, 500%) at neural plate stage (Stage 14). Stars represent statistically significant differences according to both Bonferroni-corrected two-sample Kolmogorov-Smirnov Test ($p<0.05$) and Cohen's d statistics for effect size (*: 0.2≤ ∣d∣ > 0.5, **: 0.5 ≤ ∣d∣ >0.8, ***: ∣d∣ ≥ .8). Markovian entropy was calculated with n=4 and k=1.

International Journal of *Molecular Sciences*

MDPI

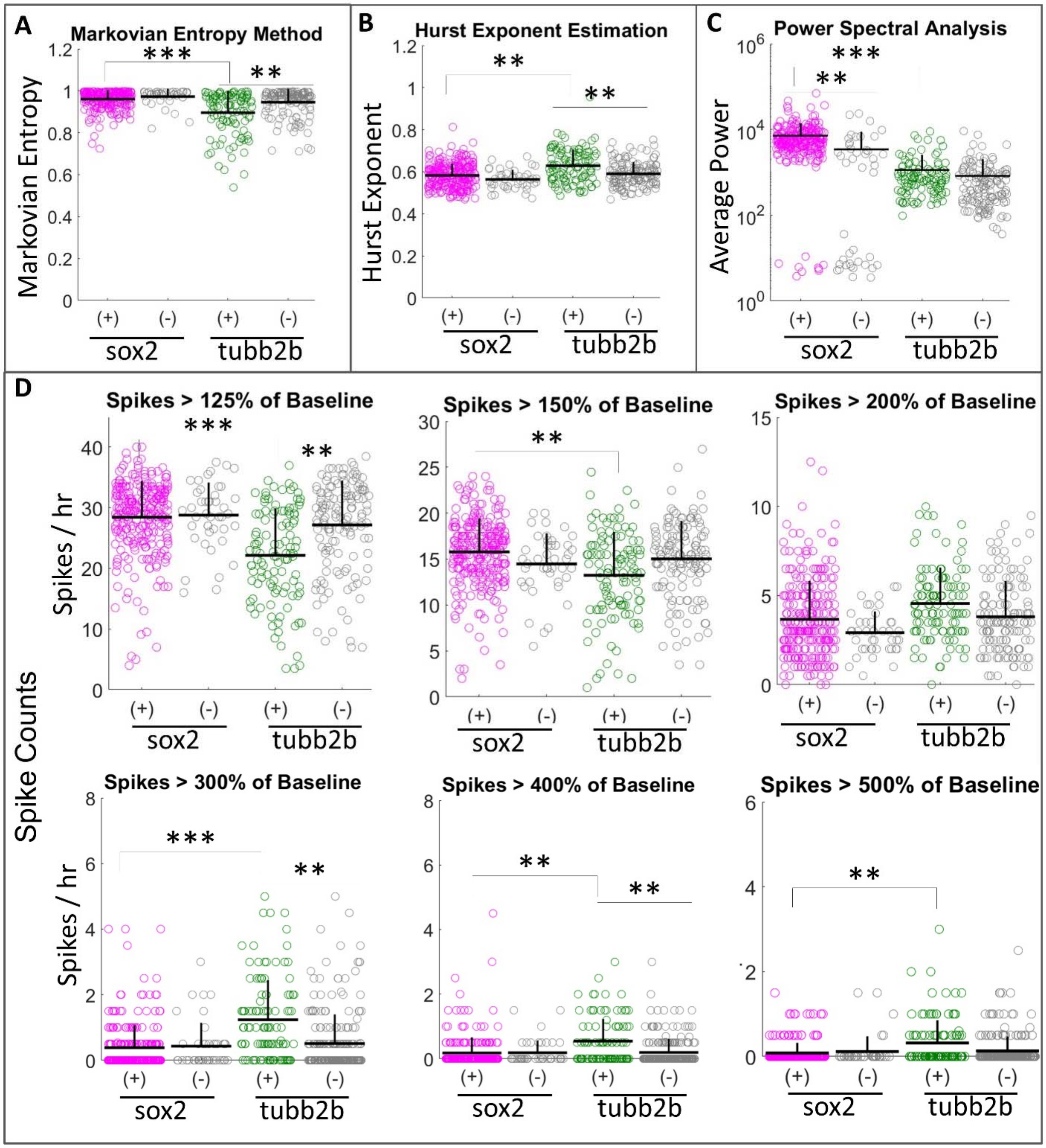

Supplementary Figure 2: Comparison of calcium activity between neural progenitors (*sox2*-positive), cells that were not *sox2* positive, differentiated (t*ubb2b*-positive) neurons, and *tubb2b*-negative cells (cells with a FISH signal intensity 200% or more than five lowest cells on the same experimental plate were considered positive for expression of the gene of interest). Calcium activity was quantified using (A) Markovian Entropy Measure (B) Hurst Exponent (C) Average power (D) Spike frequency counted at different thresholds (125%, 150%, 200%, 300%, 400%, 500%) at neural plate stage (Stage 14). Stars represent statistically significant differences according to both Bonferroni-corrected two-sample Kolmogorov-Smirnov Test ($p<0.05$) and Cohen's d statistics for effect size (*: 0.2≤ |d| > 0.5, **: 0.5 ≤ |d| >0.8, ***: |d| ≥ .8). Markovian entropy was calculated with n=4 and k=1.

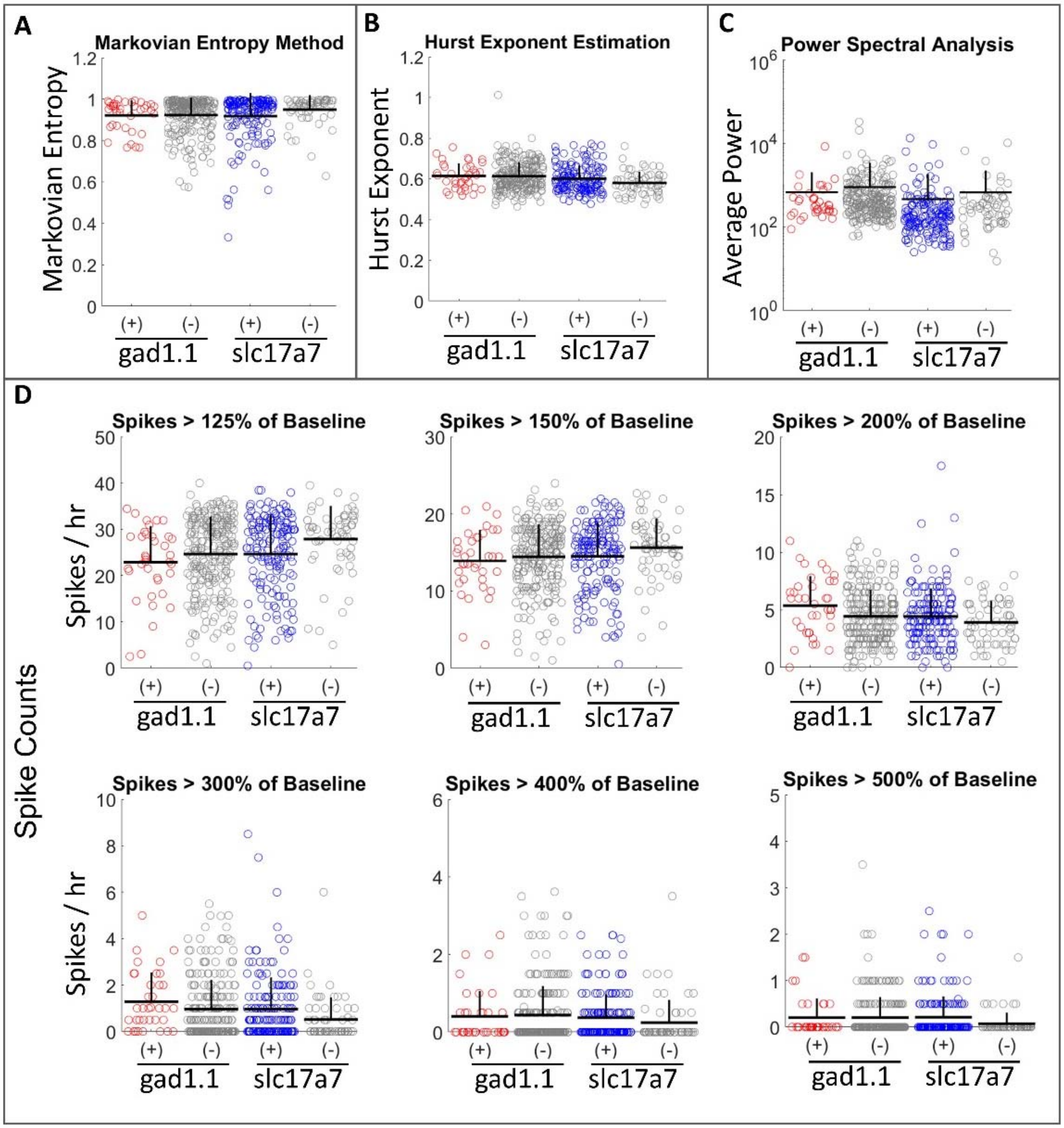

Supplementary Figure 3: Comparison of calcium activity between inhibitory (*gad1.1*-positive) neurons, *gad1.1*-negative cells, excitatory (*slc17a7*-positive) neurons, and *slc17a7*-negative cells (cells with a FISH signal intensity 300% or more than the five lowest cells on the same experimental plate were considered positive for expression of the gene of interest). Calcium activity was quantified using (A) Markovian Entropy Measure (B) Hurst Exponent (C) Average power (D) Spike frequency counted at different thresholds (125%, 150%, 200%, 300%, 400%, 500%) at neural plate stage (Stage 14). Stars represent statistically significant differences according to both Bonferroni-corrected two-sample Kolmogorov-Smirnov Test ($p<0.05$) and Cohen's d statistics for effect size (*: 0.2≤ |d| > 0.5, **: 0.5 ≤ |d| >0.8, ***: |d| ≥ .8). Markovian entropy was calculated with n=4 and k=1.

International Journal of
Molecular Sciences

MDPI

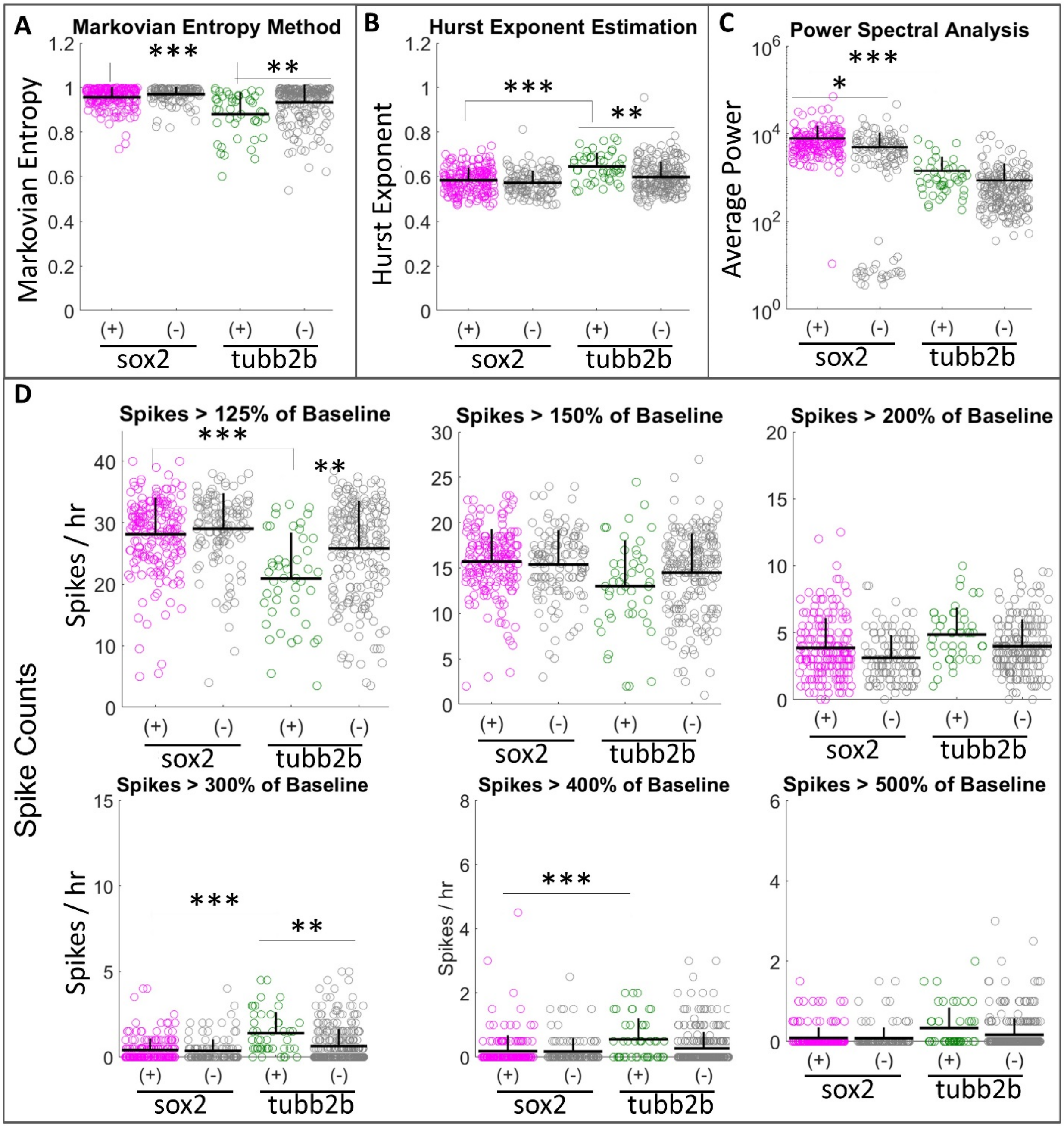

Supplementary Figure 4: Comparison of calcium activity between neural progenitors (*sox2*-positive), cells that were not *sox2*-positive, differentiated (*tubb2b*-positive) neurons, and *tubb2b*-negative cells (cells with a FISH signal intensity 300% or more than the five lowest cells on the same experimental plate were considered positive for expression of the gene of interest). Calcium activity was quantified using (A) Markovian Entropy Measure (B) Hurst Exponent (C) Average power (D) Spike frequency counted at different thresholds (125%, 150%, 200%, 300%, 400%, 500%) at neural plate stage (Stage 14). Stars represent statistically significant differences according to both Bonferroni-corrected two-sample Kolmogorov-Smirnov Test ($p<0.05$) and Cohen's d statistics for effect size (*: $0.2 \leq |d| > 0.5$, **: $0.5 \leq |d| > 0.8$, ***: $|d| \geq .8$). Markovian entropy was calculated with n=4 and k=1.

International Journal of *Molecular Sciences*

MDPI

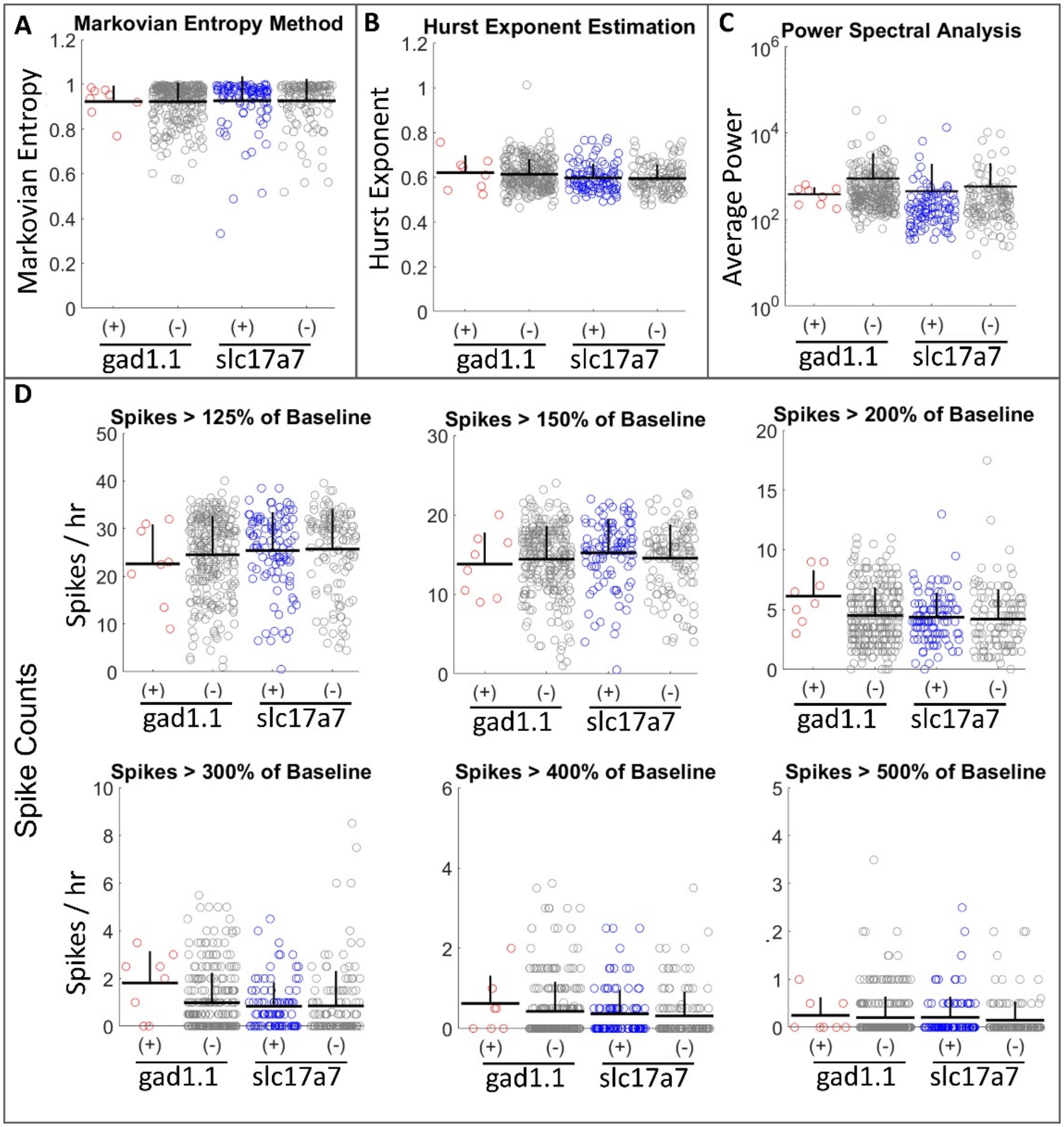

Supplementary Figure 5: Comparison of calcium activity between inhibitory (*gad1.1*-positive) neurons, *gad1.1*-negative cells, excitatory (*slc17a7*-positive) neurons, and *slc17a7*-negative cells (cells with a FISH signal intensity 400% or more than the five lowest cells on the same experimental plate were considered positive for expression of the gene of interest). Calcium activity was quantified using (A) Markovian Entropy Measure (B) Hurst Exponent (C) Average power (D) Spike frequency counted at different thresholds (125%, 150%, 200%, 300%, 400%, 500%) at neural plate stage (Stage 14). Stars represent statistically significant differences according to both Bonferroni-corrected two-sample Kolmogorov-Smirnov Test ($p<0.05$) and Cohen's d statistics for effect size (*: $0.2 \leq |d| > 0.5$, **: $0.5 \leq |d| > 0.8$, ***: $|d| \geq .8$). Markovian entropy was calculated with n=4 and k=1.

International Journal of
Molecular Sciences

MDPI

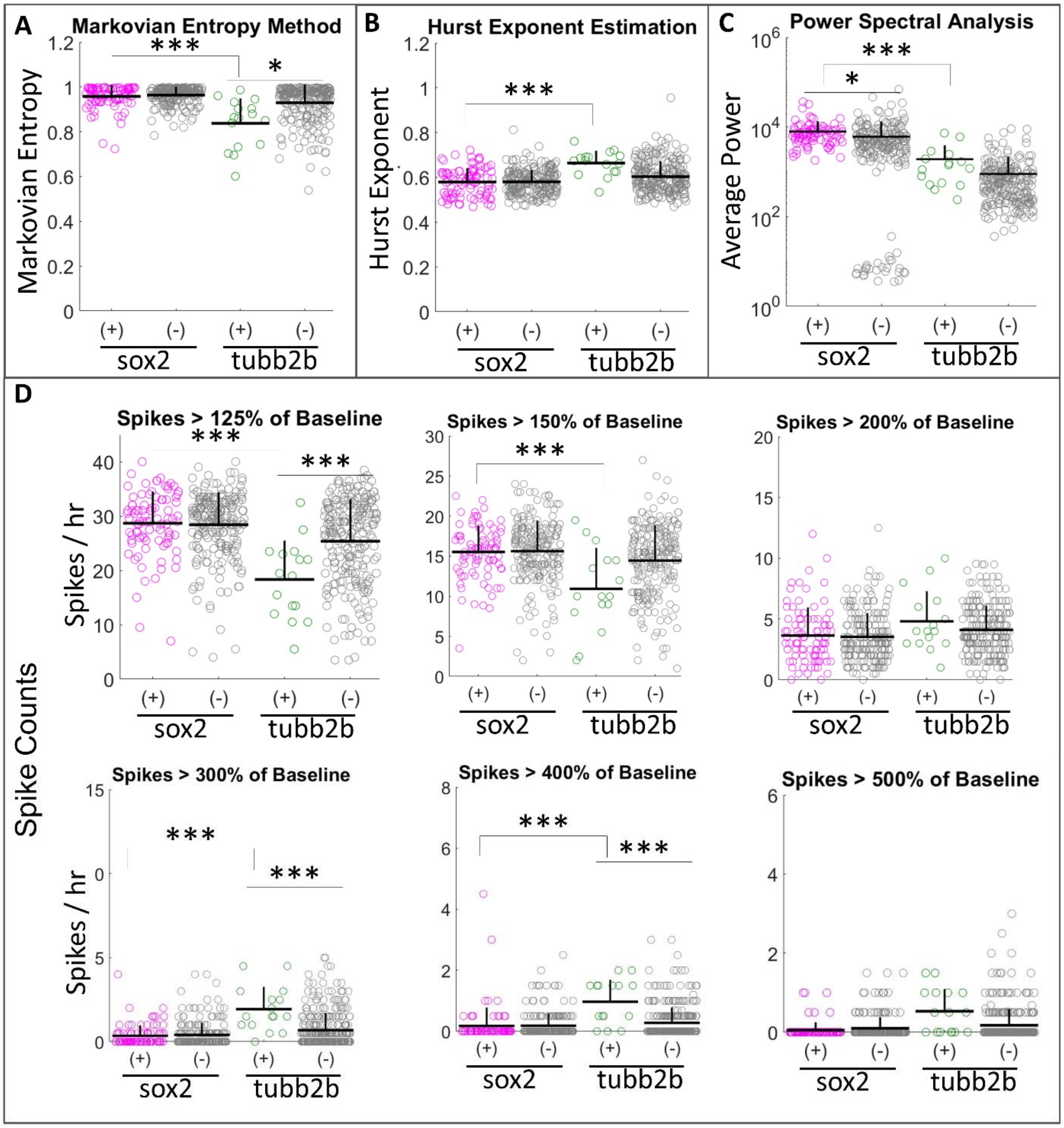

Supplementary Figure 6: Comparison of calcium activity between neural progenitors (*sox2*-positive), cells that were not *sox2*-positive, differentiated (t*ubb2b*-positive) neurons, and *tubb2b*-negative cells (cells with a FISH signal intensity 400% or more than the five lowest cells on the same experimental plate were considered positive for expression of the gene of interest). Calcium activity was quantified using (A) Markovian Entropy Measure (B) Hurst Exponent (C) Average power (D) Spike frequency counted at different thresholds (125%, 150%, 200%, 300%, 400%, 500%) at neural plate stage (Stage 14). Stars represent statistically significant differences according to both Bonferroni-corrected two-sample Kolmogorov-Smirnov Test ($p<0.05$) and Cohen's d statistics for effect size (*: $0.2 \leq |d| > 0.5$, **: $0.5 \leq |d| > 0.8$, ***: $|d| \geq .8$). Markovian entropy was calculated with n=4 and k=1.

International Journal of Molecular Sciences

MDPI

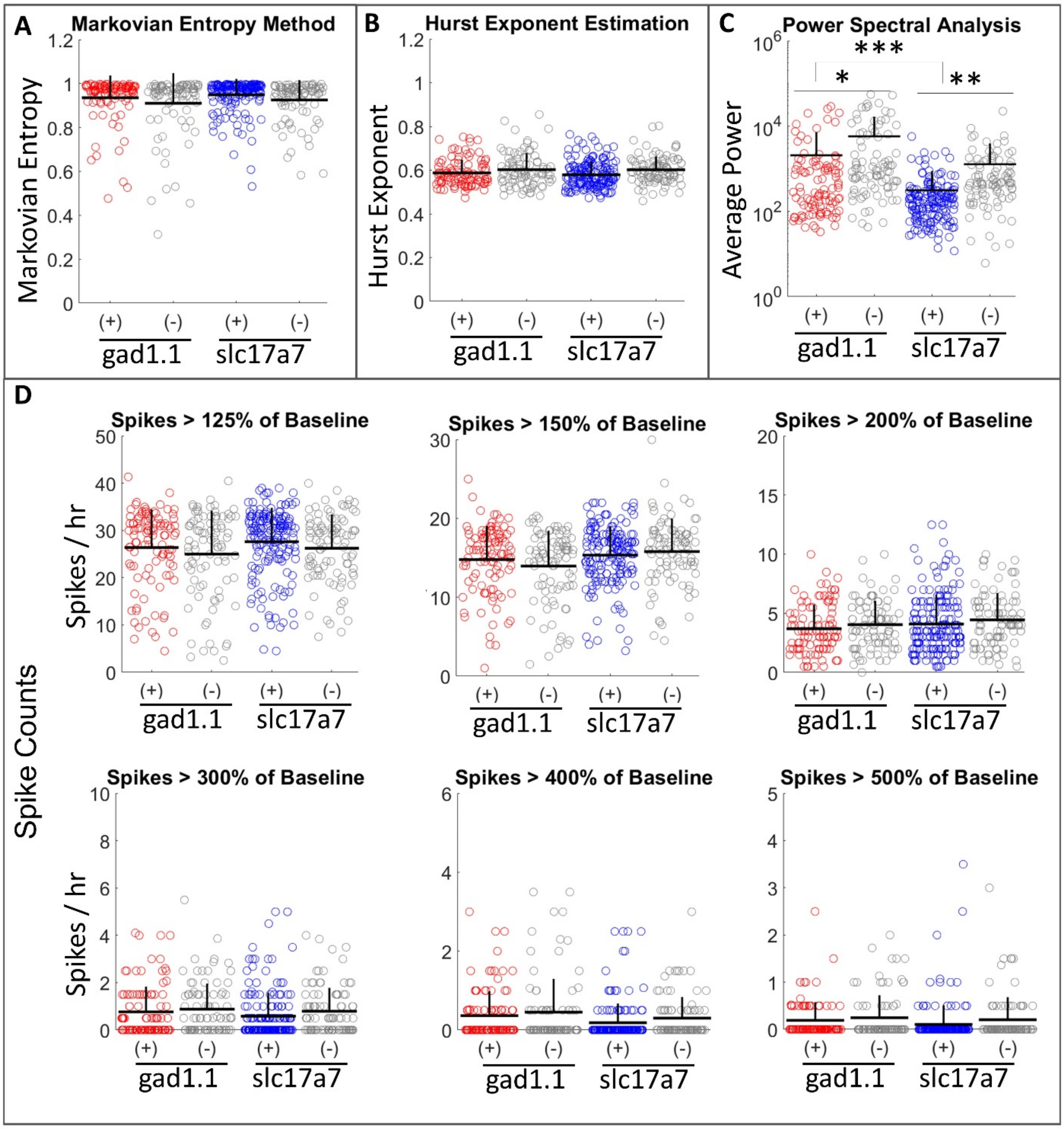

Supplementary Figure 7: Comparison of calcium activity between inhibitory (*gad1.1*-positive) neurons, *gad1.1*-negative cells, excitatory (*slc17a7*-positive) neurons, and *slc17a7*-negative cells (cells with a FISH signal intensity 200% or more than the five lowest cells on the same experimental plate were considered positive for expression of the gene of interest). Calcium activity was quantified using (A) Markovian Entropy Measure (B) Hurst Exponent (C) Average power (D) Spike frequency counted at different thresholds (125%, 150%, 200%, 300%, 400%, 500%) at neural tube stage (Stage 18). Stars represent statistically significant differences according to both Bonferroni-corrected two-sample Kolmogorov-Smirnov Test ($p<0.05$) and Cohen's d statistics for effect size (*: 0.2≤ |d| > 0.5, **: 0.5 ≤ |d| >0.8, ***: |d| ≥ .8). Markovian entropy was calculated with n=4 and k=1.

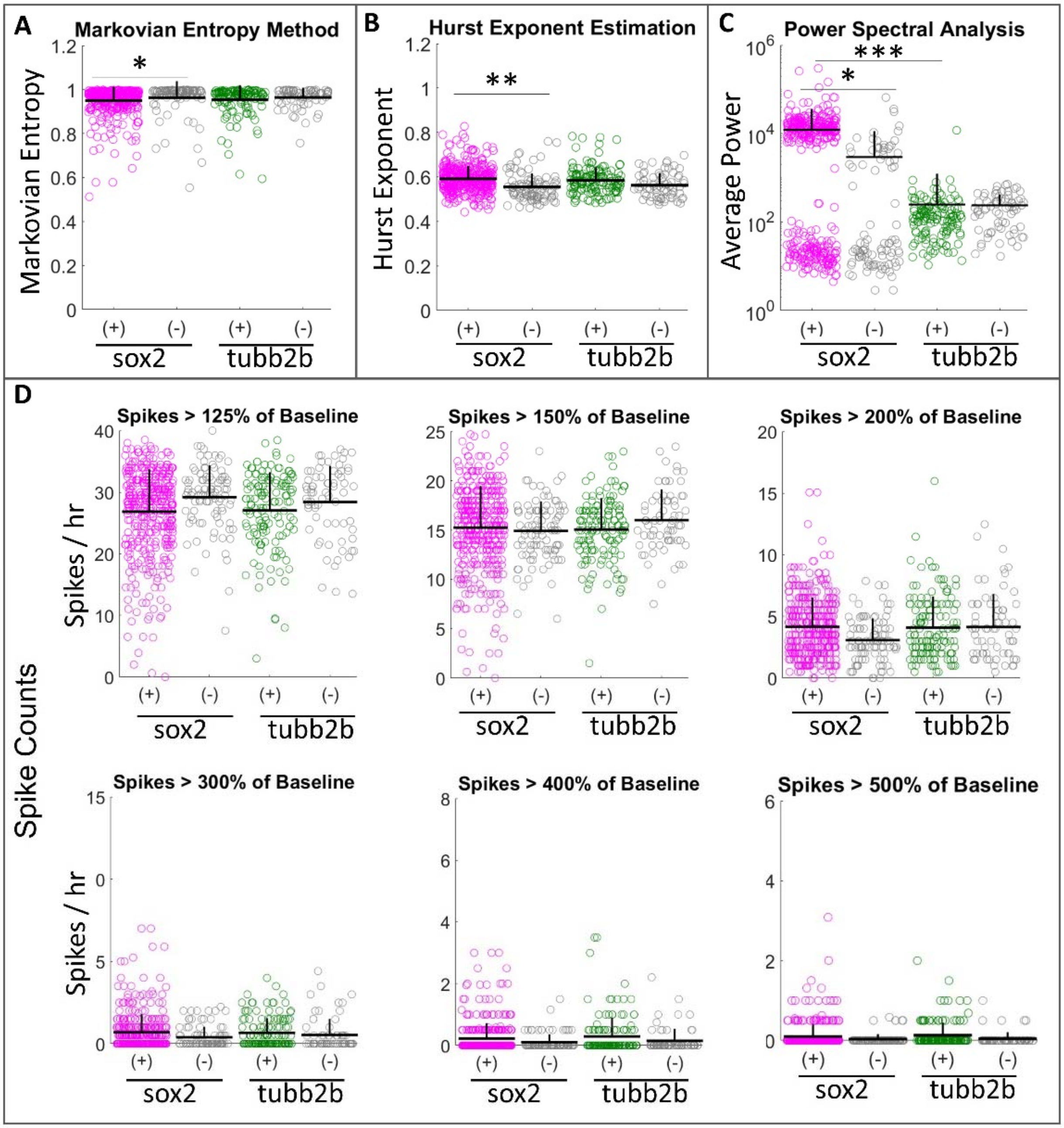

Supplementary Figure 8: Comparison of calcium activity between neural progenitors (*sox2*-positive), cells that were not *sox2*-positive, differentiated (*tubb2b*-positive) neurons, and *tubb2b*-negative cells (cells with a FISH signal intensity 200% or more than the five lowest cells on the same experimental plate were considered positive for expression of the gene of interest). Calcium activity was quantified using (A) Markovian Entropy Measure (B) Hurst Exponent (C) Average power (D) Spike frequency counted at different thresholds (125%, 150%, 200%, 300%, 400%, 500%) at neural tube stage (Stage 18). Stars represent statistically significant differences according to both Bonferroni-corrected two-sample Kolmogorov-Smirnov Test ($p<0.05$) and Cohen's d statistics for effect size (*: $0.2\leq |d| > 0.5$, **: $0.5 \leq |d| > 0.8$, ***: $|d| \geq .8$). Markovian entropy was calculated with n=4 and k=1.

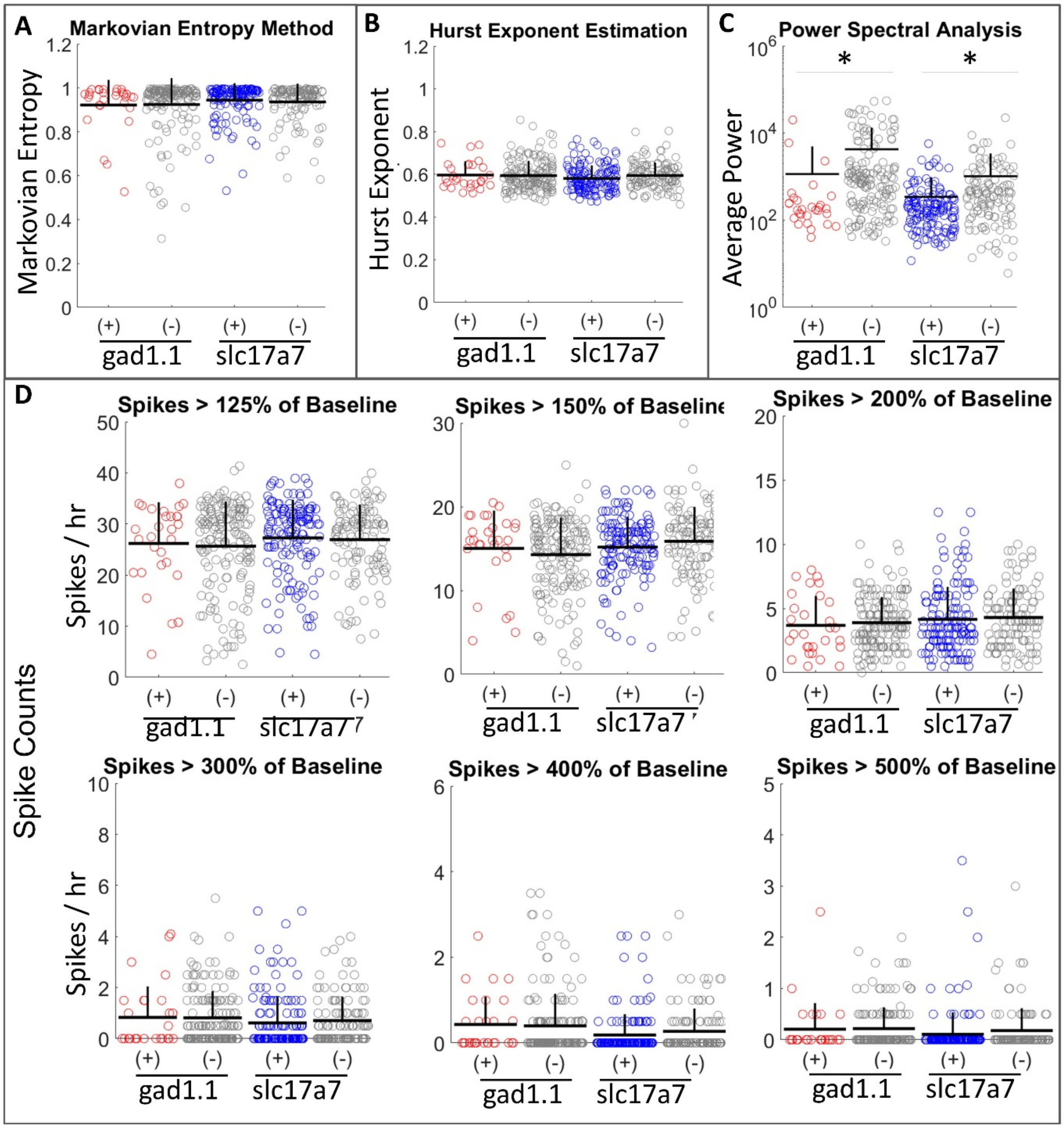

Supplementary Figure 9: Comparison of calcium activity between inhibitory (*gad1.1*-positive) neurons, *gad1.1*-negative cells, excitatory (*slc17a7*-positive) neurons, and *slc17a7*-negative cells (cells with a FISH signal intensity 300% or more than the five lowest cells on the same experimental plate were considered positive for expression of the gene of interest). Calcium activity was quantified using (A) Markovian Entropy Measure (B) Hurst Exponent (C) Average power (D) Spike frequency counted at different thresholds (125%, 150%, 200%, 300%, 400%, 500%) at neural tube stage (Stage 18). Stars represent statistically significant differences according to both Bonferroni-corrected two-sample Kolmogorov-Smirnov Test ($p<0.05$) and Cohen's d statistics for effect size (*: $0.2 \le |d| > 0.5$, **: $0.5 \le |d| > 0.8$, ***: $|d| \ge .8$). Markovian entropy was calculated with n=4 and k=1.

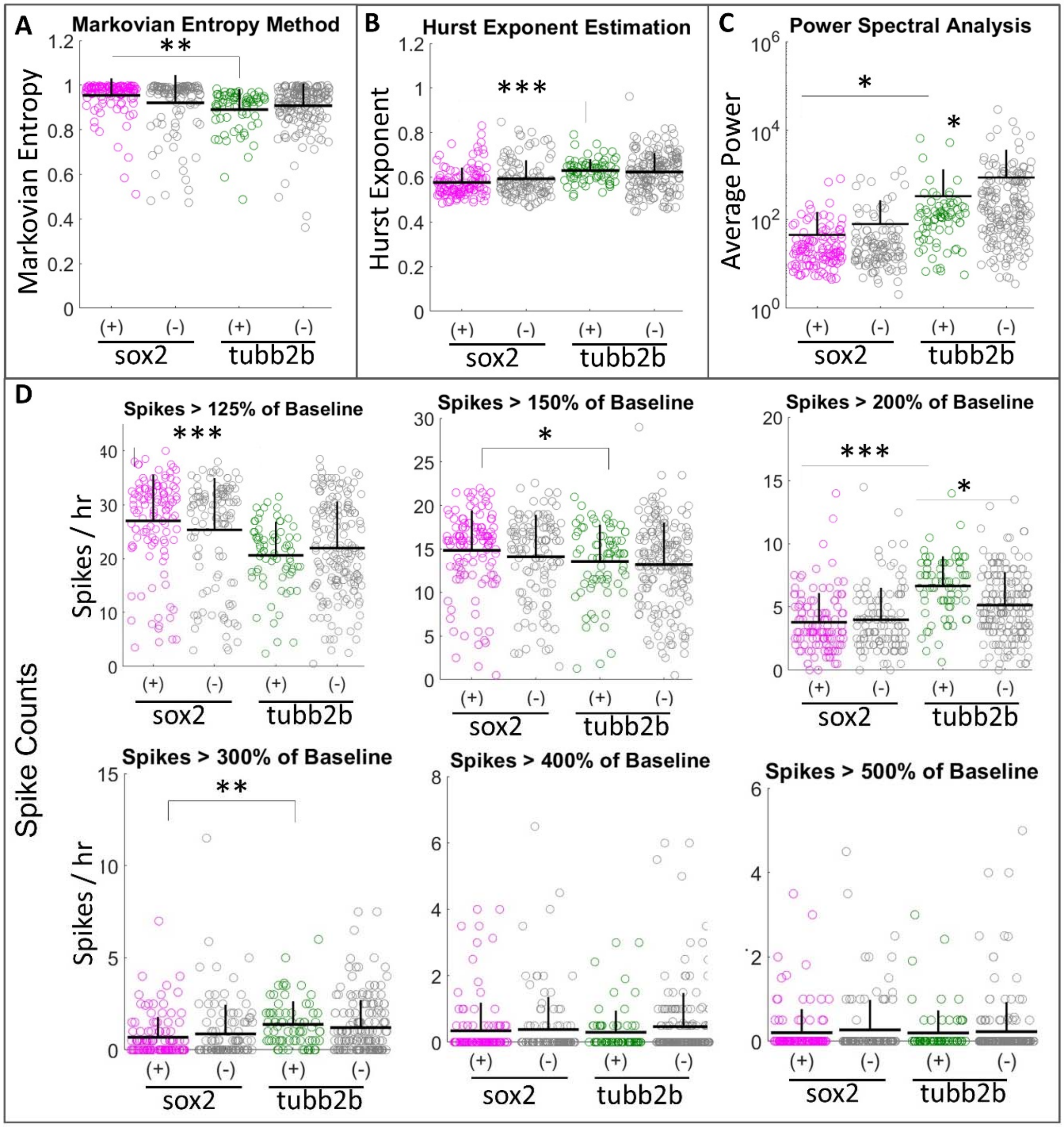

Supplementary Figure 10: Comparison of calcium activity between neural progenitors (*sox2*-positive), cells that were not *sox2*-positive, differentiated (*tubb2b*-positive) neurons, and *tubb2b*-negative cells (cells with a FISH signal intensity 300% or more than the five lowest cells on the same experimental plate were considered positive for expression of the gene of interest). Calcium activity was quantified using (A) Markovian Entropy Measure (B) Hurst Exponent (C) Average power (D) Spike frequency counted at different thresholds (125%, 150%, 200%, 300%, 400%, 500%) at neural tube stage (Stage 18). Stars represent statistically significant differences according to both Bonferroni-corrected two-sample Kolmogorov-Smirnov Test ($p<0.05$) and Cohen's d statistics for effect size (*: 0.2≤ |d| > 0.5, **: 0.5 ≤ |d| >0.8, ***: |d| ≥ .8). Markovian entropy was calculated with n=4 and k=1.

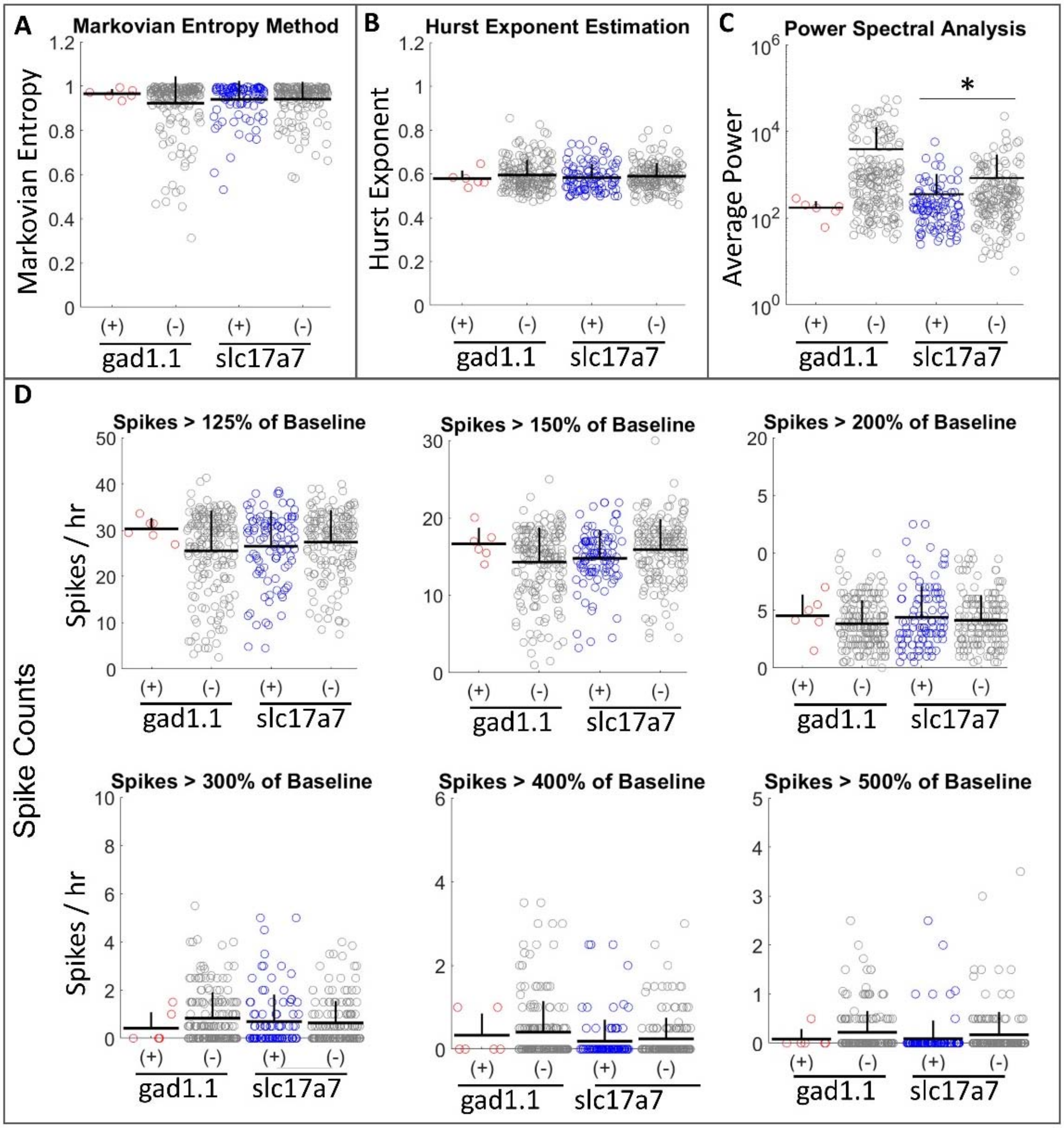

Supplementary Figure 11: Comparison of calcium activity between inhibitory (*gad1.1*-positive) neurons, *gad1.1*-negative cells, excitatory (*slc17a7*)-positive neurons, and *slc17a7*-negative cells (cells with a FISH signal intensity 400% or more than the five lowest cells on the same experimental plate were considered positive for expression of the gene of interest). Calcium activity was quantified using (A) Markovian Entropy Measure (B) Hurst Exponent (C) Average power (D) Spike frequency counted at different thresholds (125%, 150%, 200%, 300%, 400%, 500%) at neural tube stage (Stage 18). Stars represent statistically significant differences according to both Bonferroni-corrected two-sample Kolmogorov-Smirnov Test ($p<0.05$) and Cohen's d statistics for effect size (*: 0.2≤ |d| > 0.5, **: 0.5 ≤ |d| >0.8, ***: |d| ≥ .8). Markovian entropy was calculated with n=4 and k=1.

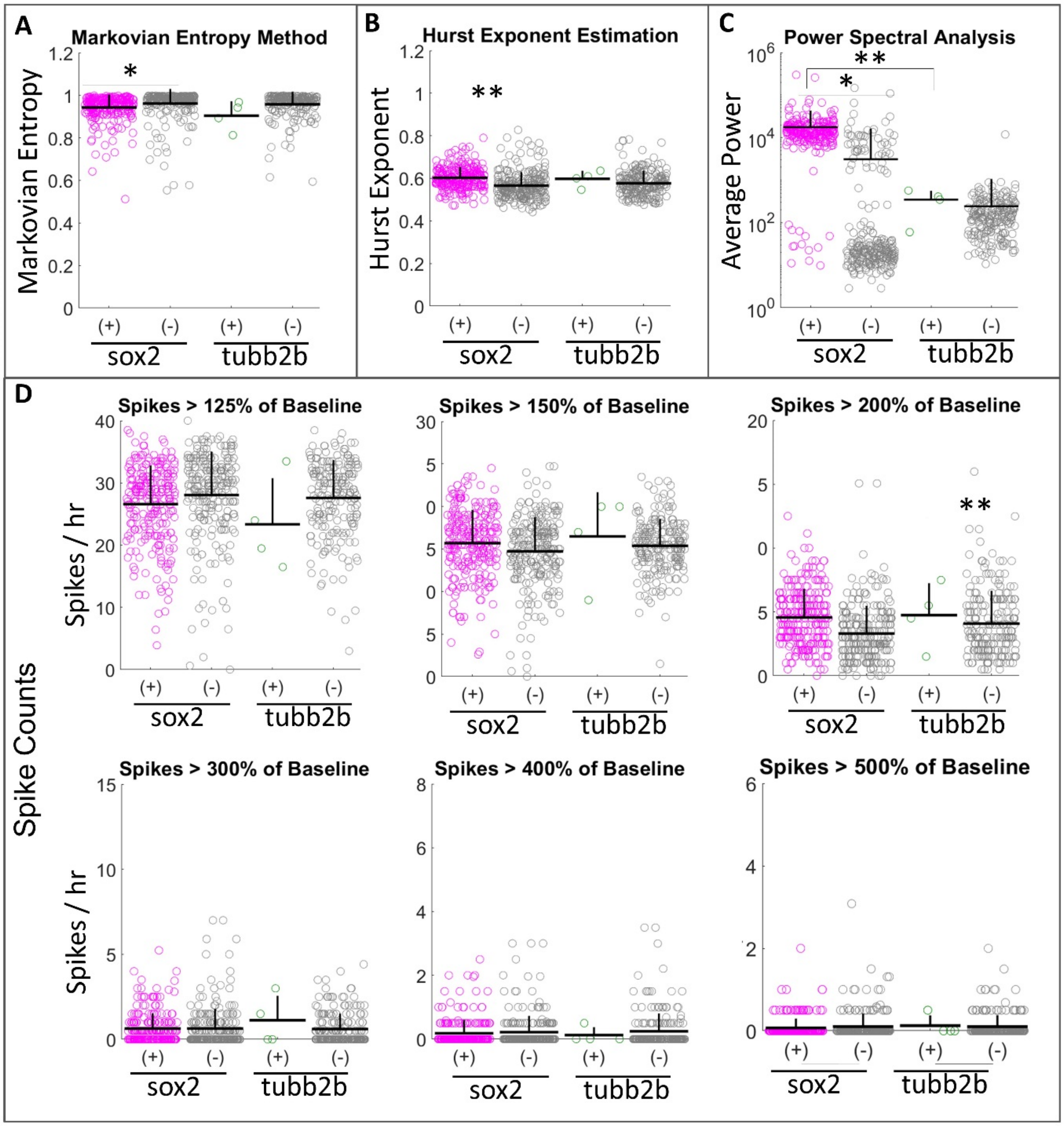

Supplementary Figure 12: Comparison of calcium activity between neural progenitors (*sox2*-positive), cells that were not *sox2*-positive, differentiated (*tubb2b*-positive) neurons, and *tubb2b*-negative cells (cells with a FISH signal intensity 400% or more than the five lowest cells on the same experimental plate were considered positive for expression of the gene of interest). Calcium activity was quantified using (A) Markovian Entropy Measure (B) Hurst Exponent (C) Average power (D) Spike frequency counted at different thresholds (125%, 150%, 200%, 300%, 400%, 500%) at neural tube stage (Stage 18). Stars represent statistically significant differences according to both Bonferroni-corrected two-sample Kolmogorov-Smirnov Test ($p<0.05$) and Cohen's d statistics for effect size (*: $0.2\leq |d| > 0.5$, **: $0.5 \leq |d| >0.8$, ***: $|d| \geq .8$). Markovian entropy was calculated with n=4 and k=1.

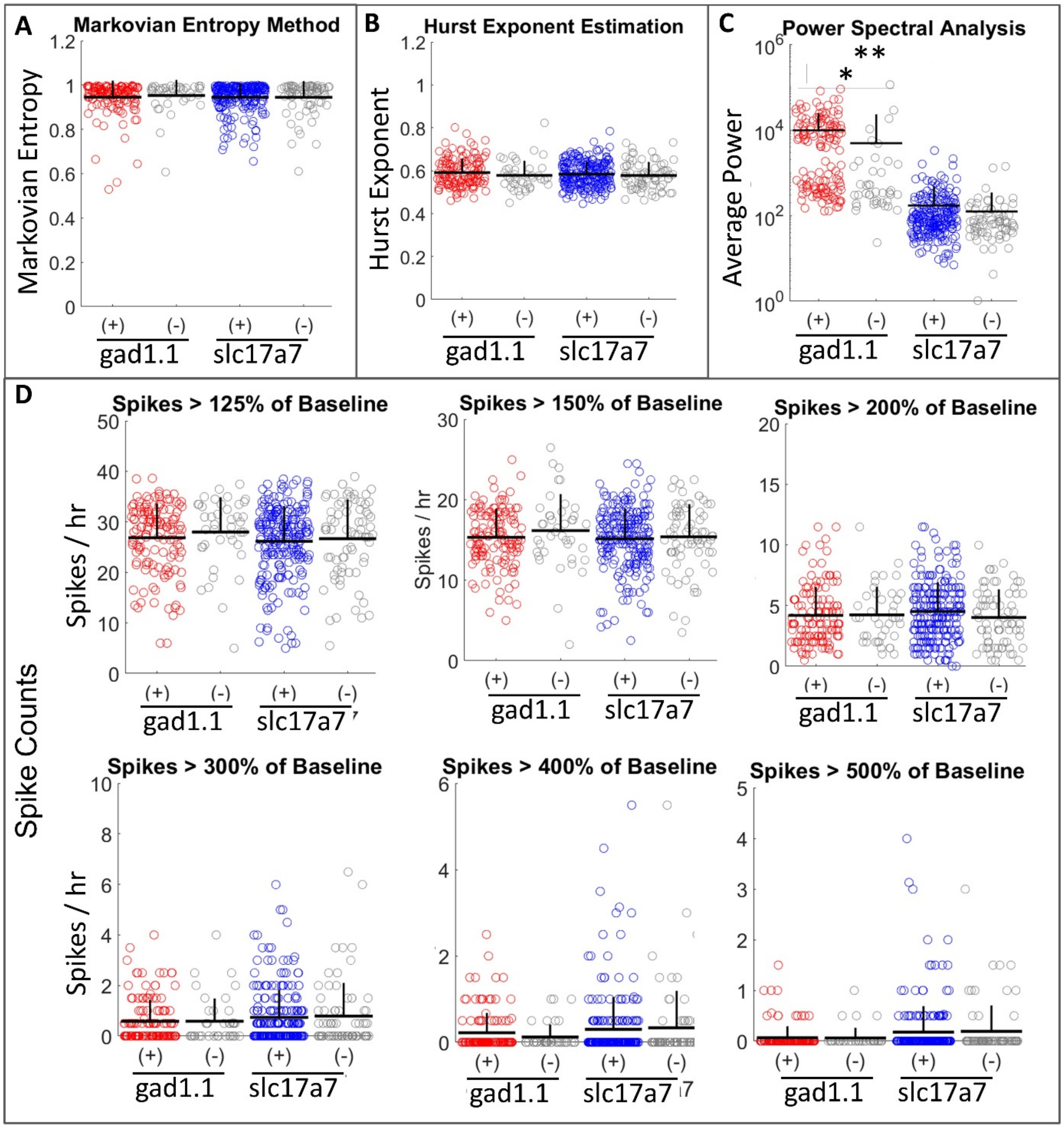

Supplementary Figure 13: Comparison of calcium activity between inhibitory (*gad1.1*-positive) neurons, *gad1.1*-negative cells, excitatory (*slc17a7*)-positive neurons, and *slc17a7*-negative cells (cells with a FISH signal intensity 200% or more than the five lowest cells on the same experimental plate were considered positive for expression of the gene of interest). Calcium activity was quantified using (A) Markovian Entropy Measure (B) Hurst Exponent (C) Average power (D) Spike frequency counted at different thresholds (125%, 150%, 200%, 300%, 400%, 500%) at early tail bud stage (Stage 22). Stars represent statistically significant differences according to both Bonferroni-corrected two-sample Kolmogorov-Smirnov Test ($p<0.05$) and Cohen's d statistics for effect size (*: 0.2≤ |d| > 0.5, **: 0.5 ≤ |d| >0.8, ***: |d| ≥ .8). Markovian entropy was calculated with n=4 and k=1.

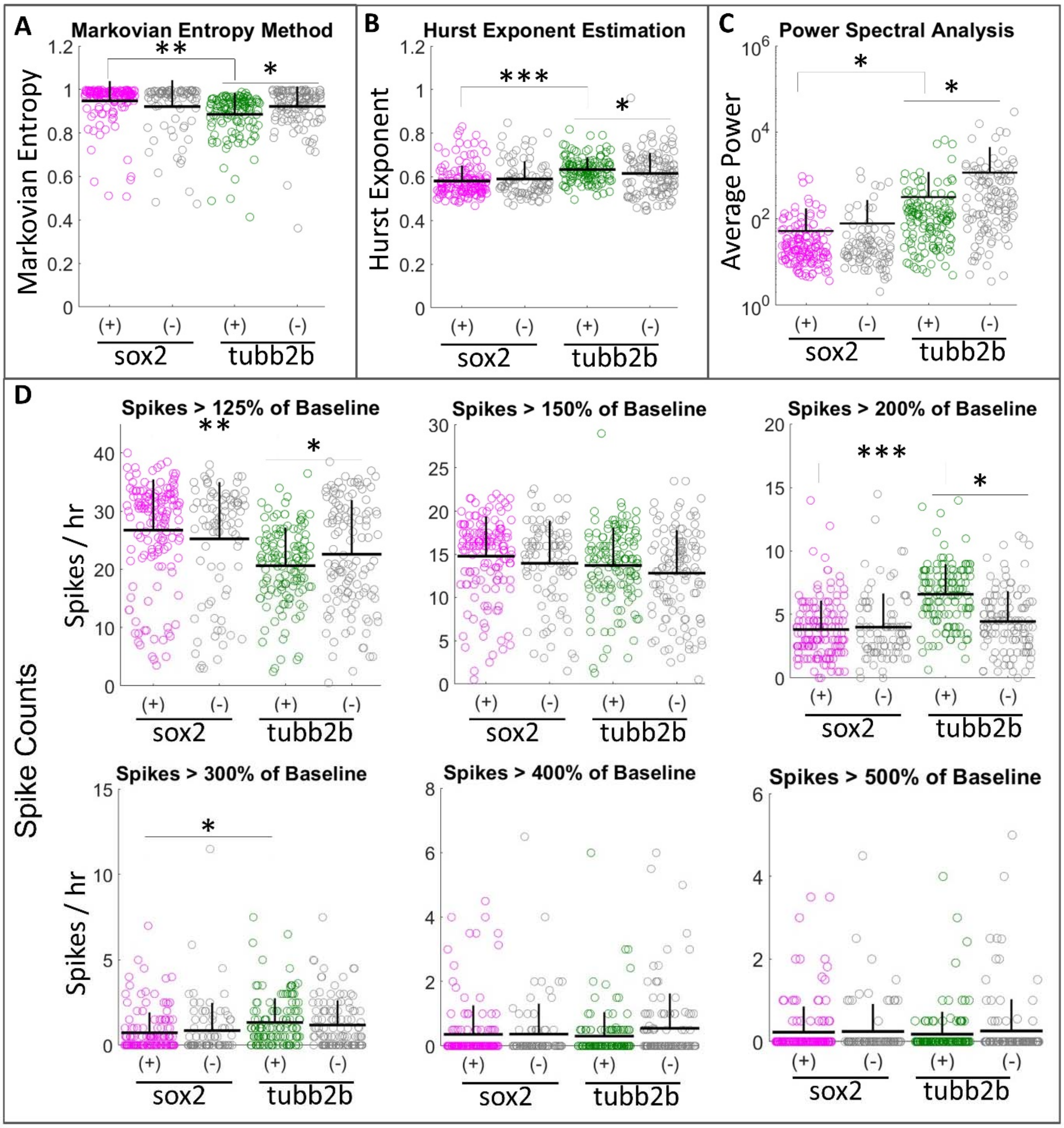

Supplementary Figure 14: Comparison of calcium activity between neural progenitors (*sox2*-positive), cells that were not *sox2*-positive, differentiated (*tubb2b*-positive) neurons, and *tubb2b*-negative cells (cells with a FISH signal intensity 200% or more than the five lowest cells on the same experimental plate were considered positive for expression of the gene of interest). Calcium activity was quantified using (A) Markovian Entropy Measure (B) Hurst Exponent (C) Average power (D) Spike frequency counted at different thresholds (125%, 150%, 200%, 300%, 400%, 500%) at early tail bud stage (Stage 22). Stars represent statistically significant differences according to both Bonferroni-corrected two-sample Kolmogorov-Smirnov Test ($p<0.05$) and Cohen's d statistics for effect size (*: 0.2≤ |d| > 0.5, **: 0.5 ≤ |d| >0.8, ***: |d| ≥ .8). Markovian entropy was calculated with n=4 and k=1.

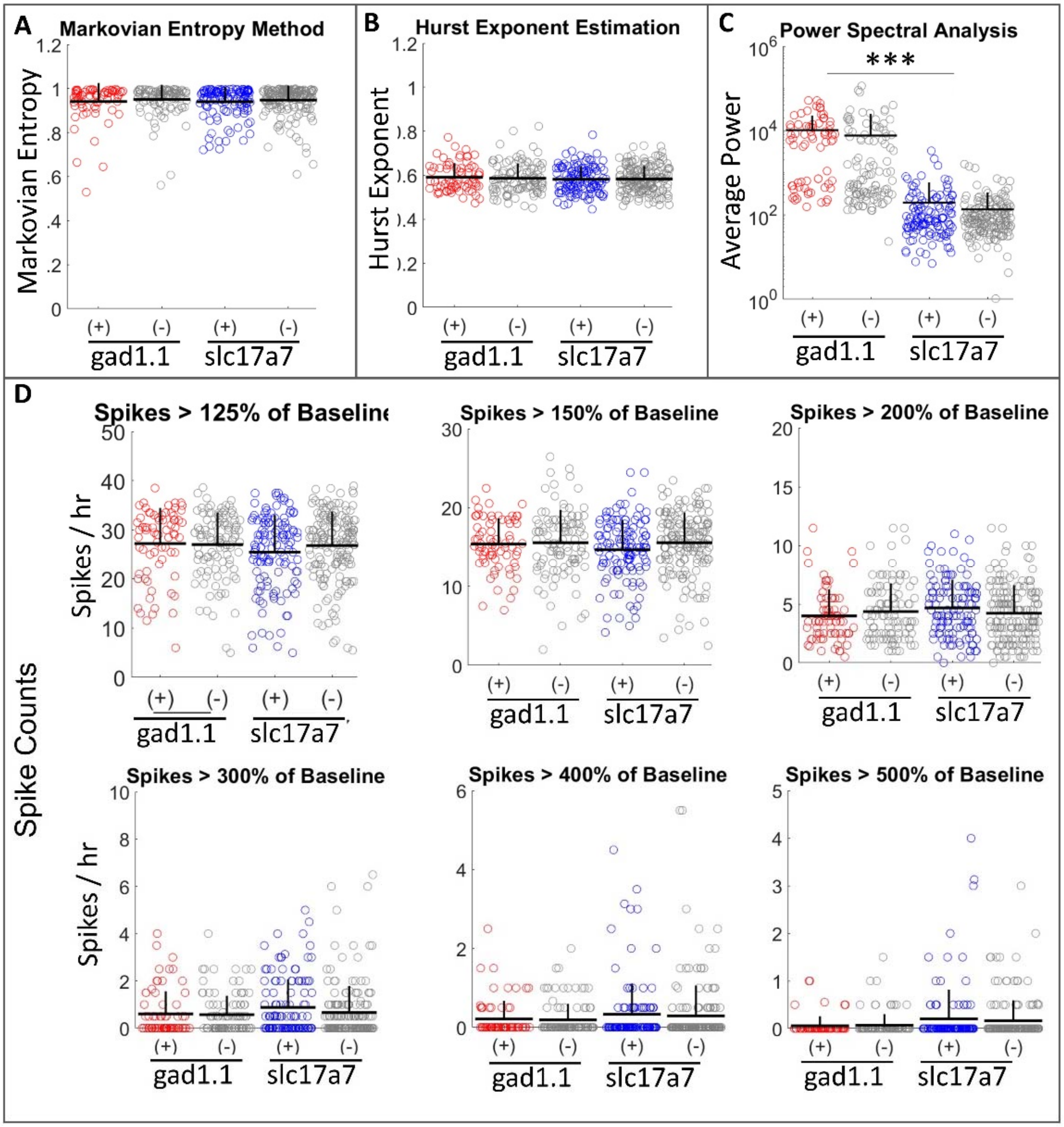

Supplementary Figure 15: Comparison of calcium activity between inhibitory (*gad1.1*-positive) neurons, *gad1.1*-negative cells, excitatory (*slc17a7*)-positive neurons, and *slc17a7*-negative cells (cells with a FISH signal intensity 300% or more than the five lowest cells on the same experimental plate were considered positive for expression of the gene of interest). Calcium activity was quantified using (A) Markovian Entropy Measure (B) Hurst Exponent (C) Average power (D) Spike frequency counted at different thresholds (125%, 150%, 200%, 300%, 400%, 500%) at early tail bud stage (Stage 22). Stars represent statistically significant differences according to both Bonferroni-corrected two-sample Kolmogorov-Smirnov Test ($p<0.05$) and Cohen's d statistics for effect size (*: 0.2≤ |d| > 0.5, **: 0.5 ≤ |d| >0.8, ***: |d| ≥ .8). Markovian entropy was calculated with n=4 and k=1.

International Journal of *Molecular Sciences*

MDPI

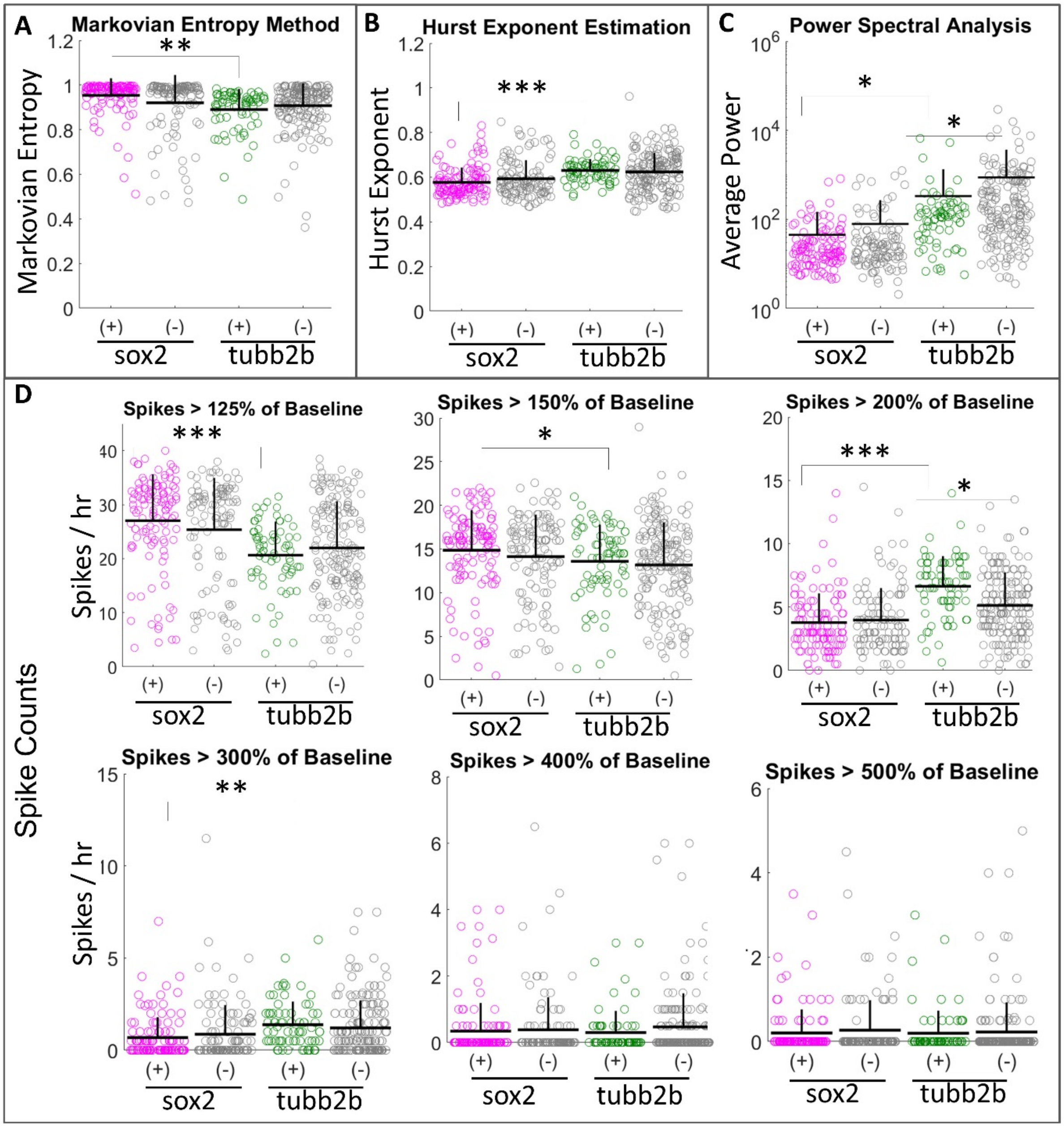

Supplementary Figure 16: Comparison of calcium activity between neural progenitors (*sox2*-positive), cells that were not *sox2*-positive, differentiated (*tubb2b*-positive) neurons, and *tubb2b*-negative cells (cells with a FISH signal intensity 300% or more than the five lowest cells on the same experimental plate were considered positive for expression of the gene of interest). Calcium activity was quantified using (A) Markovian Entropy Measure (B) Hurst Exponent (C) Average power (D) Spike frequency counted at different thresholds (125%, 150%, 200%, 300%, 400%, 500%) at early tail bud stage (Stage 22). Stars represent statistically significant differences according to both Bonferroni-corrected two-sample Kolmogorov-Smirnov Test ($p<0.05$) and Cohen's d statistics for effect size (*: 0.2≤ |d| > 0.5, **: 0.5 ≤ |d| >0.8, ***: |d| ≥ .8). Markovian entropy was calculated with n=4 and k=1.

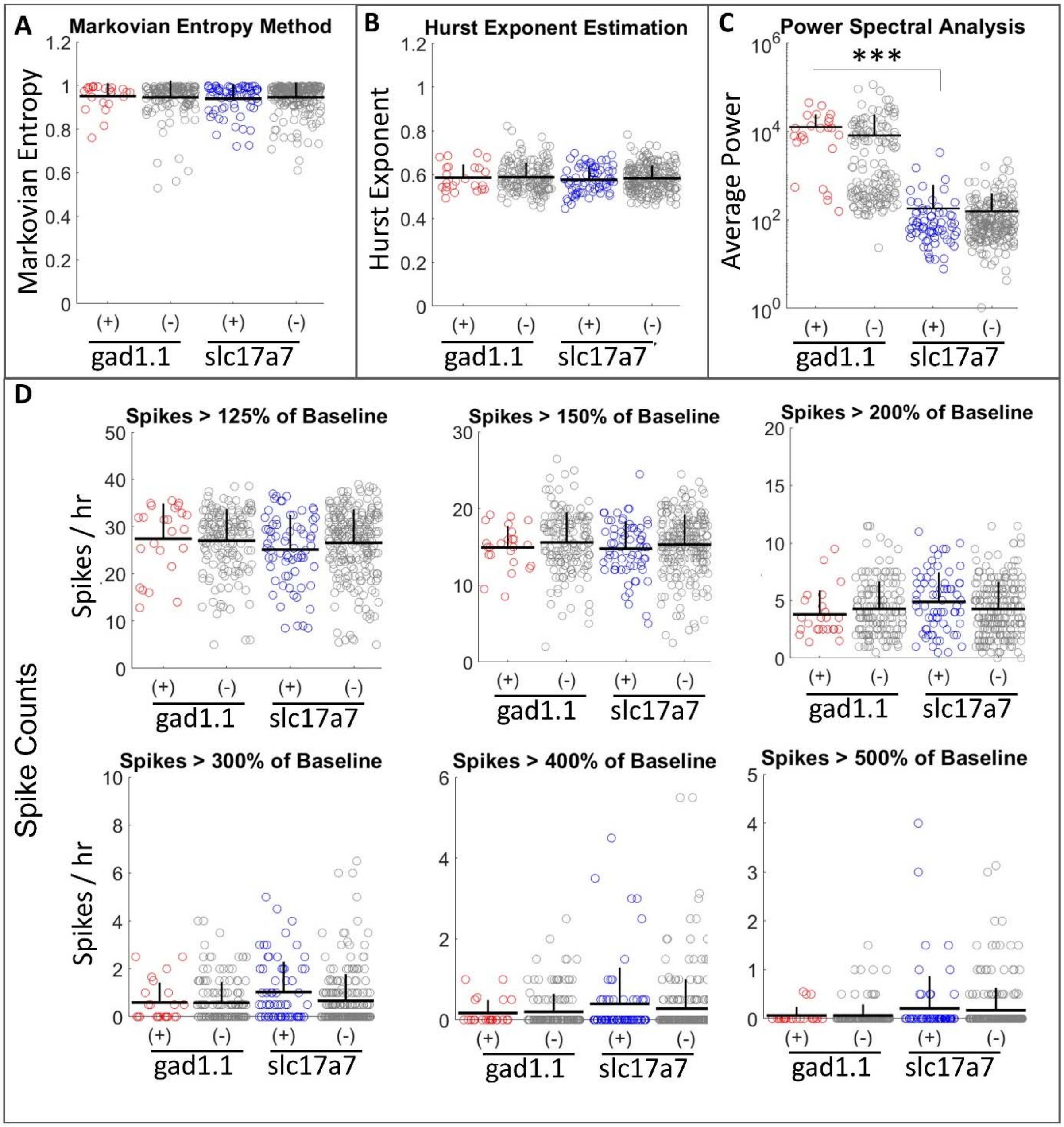

Supplementary Figure 17: Comparison of calcium activity between inhibitory (*gad1.1*-positive) neurons, *gad1.1*-negative cells, excitatory (*slc17a7*)-positive neurons, and *slc17a7*-negative cells (cells with a FISH signal intensity 400% or more than the five lowest cells on the same experimental plate were considered positive for expression of the gene of interest). Calcium activity was quantified using (A) Markovian Entropy Measure (B) Hurst Exponent (C) Average power (D) Spike frequency counted at different thresholds (125%, 150%, 200%, 300%, 400%, 500%) at early tail bud stage (Stage 22). Stars represent statistically significant differences according to both Bonferroni-corrected two-sample Kolmogorov-Smirnov Test ($p<0.05$) and Cohen's d statistics for effect size (*: 0.2≤ |d| > 0.5, **: 0.5 ≤ |d| >0.8, ***: |d| ≥ .8). Markovian entropy was calculated with n=4 and k=1.

International Journal of Molecular Sciences

MDPI

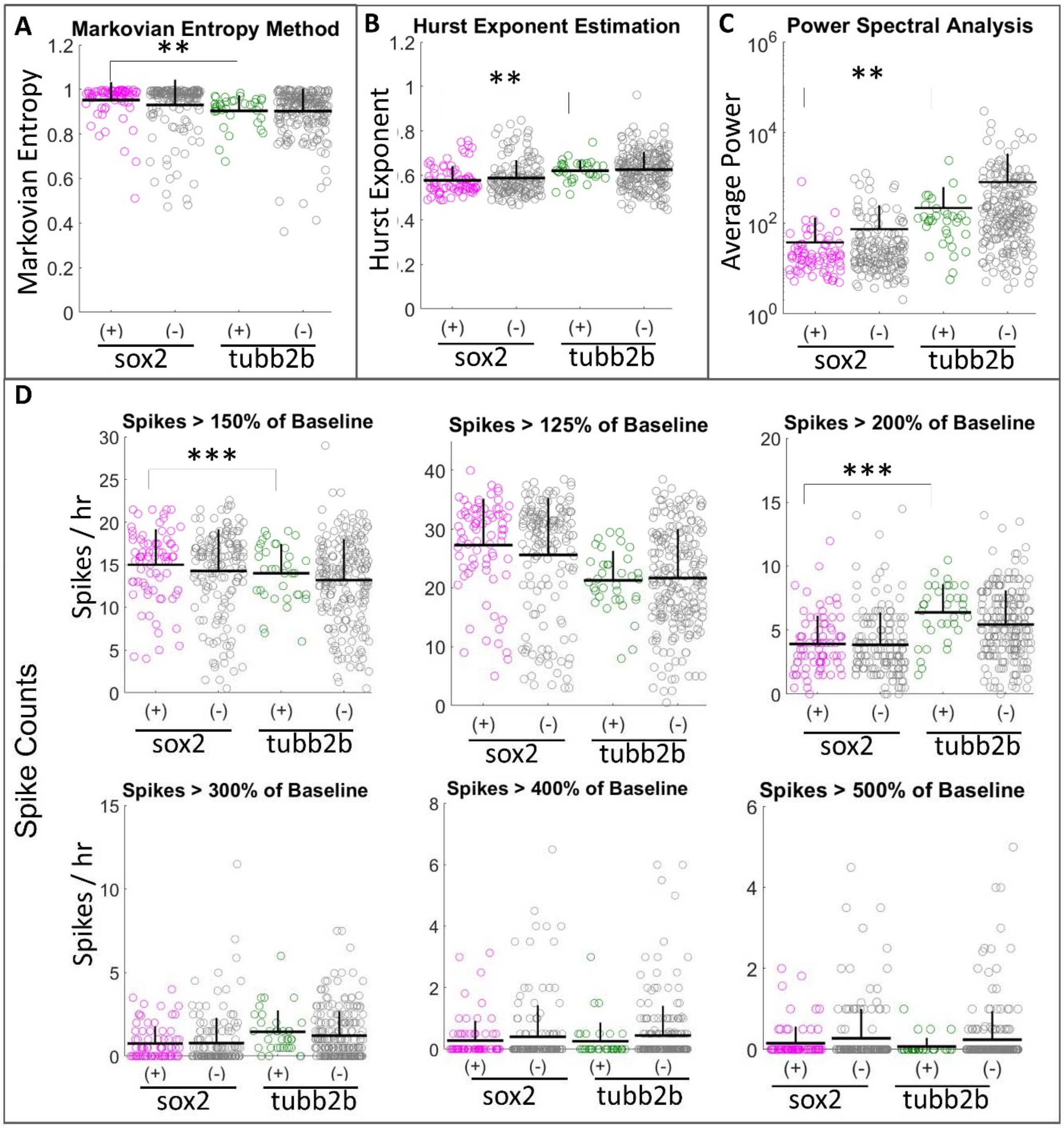

Supplementary Figure 18: Comparison of calcium activity between neural progenitors (*sox2*-positive), cells that were not *sox2*-positive, differentiated (*tubb2b*-positive) neurons, and *tubb2b*-negative cells (cells with a FISH signal intensity 400% or more than the five lowest cells on the same experimental plate were considered positive for expression of the gene of interest). Calcium activity was quantified using (A) Markovian Entropy Measure (B) Hurst Exponent (C) Average power (D) Spike frequency counted at different thresholds (125%, 150%, 200%, 300%, 400%, 500%) at early tail bud stage (Stage 22). Stars represent statistically significant differences according to both Bonferroni-corrected two-sample Kolmogorov-Smirnov Test ($p<0.05$) and Cohen's d statistics for effect size (*: 0.2≤ |d| > 0.5, **: 0.5 ≤ |d| >0.8, ***: |d| ≥ .8). Markovian entropy was calculated with n=4 and k=1.
